# STING activation in alveolar macrophages and group 2 innate lymphoid cells suppresses IL-33–driven type 2 immunopathology

**DOI:** 10.1172/jci.insight.143509

**Published:** 2021-02-08

**Authors:** Li She, Gema D. Barrera, Liping Yan, Hamad H. Alanazi, Edward G. Brooks, Peter H. Dube, Yilun Sun, Hong Zan, Daniel P. Chupp, Nu Zhang, Xin Zhang, Yong Liu, Xiao-Dong Li

**Affiliations:** 1 Department of Microbiology, Immunology and Molecular Genetics, Long School of Medicine, University of Texas Health San Antonio, San Antonio, Texas, USA.; 2 Department of Otolaryngology-Head and Neck Surgery, Xiangya Hospital, Central South University, Changsha, China.; 3 Division of Immunology and Infectious Disease, Long School of Medicine, University of Texas Health San Antonio, San Antonio, Texas, USA.; 4 Clinical Research Center for Pharyngolaryngeal Diseases and Voice Disorders,; 5 Otolaryngology Major Disease Research Key Laboratory of Hunan Province, and; 6 National Clinical Research Center for Geriatric Disorders, Xiangya Hospital, Central South University, Changsha, China.

**Keywords:** Immunology, Inflammation, Allergy, Asthma, Innate immunity

## Abstract

2′3′-cGAMP is known as a nonclassical second messenger and small immune modulator that possesses potent antitumor and antiviral activities via inducing the stimulator of IFN genes–mediated (STING-mediated) signaling pathway. However, its function in regulating type 2 immune responses remains unknown. Therefore, we sought to determine a role of STING activation by 2′3′-cGAMP in type 2 inflammatory reactions in multiple mouse models of eosinophilic asthma. We discovered that 2′3′-cGAMP administration strongly attenuated type 2 lung immunopathology and airway hyperreactivity induced by IL-33 and a fungal allergen, *Aspergillus flavus*. Mechanistically, upon the respiratory delivery, 2′3′-cGAMP was mainly internalized by alveolar macrophages, in which it activated the STING/IFN regulatory factor 3/type I IFN signaling axis to induce the production of inhibitory factors containing IFN-α, which blocked the IL-33–mediated activation of group 2 innate lymphoid (ILC2) cells in vivo. We further demonstrated that 2′3′-cGAMP directly suppressed the proliferation and function of both human and mouse ILC2 cells in vitro. Taken together, our findings suggest that STING activation by 2′3′-cGAMP in alveolar macrophages and ILC2 cells can negatively regulate type 2 immune responses, implying that the respiratory delivery of 2′3′-cGAMP might be further developed as an alternative strategy for treating type 2 immunopathologic diseases such as eosinophilic asthma.

## Introduction

Asthma is a chronic condition of airway inflammation characterized by the recurrent episodes of airway obstruction and wheezing. The prevalence and incidence of asthma are very high and still rising in the industrialized world. Clinically, asthma is a very complex and heterogenous disease comprising different subtypes. Eosinophilic asthma is the most common form featured by airway eosinophilia, elevated levels of serum IgE, and type 2 cytokines IL-4, IL-5, and IL-13. Since the primary cause and underlying mechanism of the disease remain poorly understood, current treatments can only provide a long-term control of symptoms, but fail to cure or prevent the disease (1–4).

In the past few years, it has become increasingly appreciated that group 2 innate lymphoid (ILC2) cells play a critical role in the initiation and orchestration of eosinophilic inflammation (5). Innate lymphoid cells lack rearranged antigen receptors expressed on B and T cells and are the innate counterparts of T lymphocytes (5–8). By reacting promptly to environmental signals, or type 2 inducer cytokines such as IL-33, activated ILC2 cells are capable of producing massive amounts of the type 2 cytokines IL-5 and IL-13 (9–12), which promote the development of eosinophilia, airway remodeling, and mucus hypersecretion, respectively. Additionally, ILC2 cell–derived IL-13 can potentiate memory T helper 2 cell responses by licensing dendritic cells (13). Notably, IL-33 is a new member of the IL-1 superfamily of cytokines and induces the production of type 2 cytokines in ILC2 and Th2 cells to promote the pathogenesis of many eosinophilic diseases such as asthma (9–12). Owing to their enormous capacity in instigating and amplifying type 2 inflammatory responses, activation of ILC2 cells must be tightly regulated. Indeed, emerging evidence suggests that diversified receptors expressed on the surface of ILC2 cells can either enhance or repress their activation and proliferation in response to various signals such as alarmin cytokines, hormones, regulatory cytokines, neuropeptides, and lipids (14). Recent studies have also shown that microbial ligands, including unmethylated CpG-DNA, can activate the corresponding innate immune responses to attenuate eosinophilic inflammation via inhibiting ILC2 cell function in mouse models of asthma (15–17). However, it is unclear whether other innate immune stimuli such as 2′3′-cGAMP can regulate type 2 immunity through targeting ILC2 cells. 2′3′-cGAMP was discovered as a nonclassical second messenger synthesized by the novel DNA sensor cyclic GMP-AMP synthase (cGAS) in response to invasion of cytosolic DNA when mammalian cells are infected by DNA viruses and intracellular bacteria (18–20). 2′3′-cGAMP exclusively binds and robustly activates stimulator of IFN genes (STING), which subsequently recruits the kinase TBK1 to trigger a signaling cascade leading to the production of type I IFN (IFN-I). Currently, 2′3′-cGAMP or its derivatives are being intensively investigated in a number of clinical trials for enhancing the efficacy of anti-PD-1–based cancer immunotherapies against multiple tumor types, including advanced solid tumors and lymphomas (21–23). In addition, 2′3′-cGAMP can be used as an immune adjuvant to enhance antigen-specific humoral and cellular immunities in a vaccine setting (19).

In this study, we sought to determine an immune modulatory function of 2′3′-cGAMP in regulating type 2 inflammation in multiple mouse models of eosinophilic asthma. We demonstrate that 2′3′-cGAMP triggers the robust production of IFN-I in mouse lungs and strongly suppresses both IL-33 and an environmental allergen *Aspergillus flavus*–induced type 2 lung inflammation and airway hyperreactivity (AHR). Mechanistically, the STING/IFN-I signaling mediates the inhibitory effects of 2′3′-cGAMP and seems to act directly on ILC2 cells to inhibit their proliferation and function in the cytokine production. Collectively, our results identify an innate immune-driven mechanism for the 2′3′-cGAMP/STING/IFN-I signaling in regulating ILC2 cell function and show the potential development of this new mammalian cyclic dinucleotide for the prevention and treatment of eosinophilic asthma.

## Results

### 2′3′-cGAMP inhibits IL-33–induced type 2 immunopathology.

**Figure 1:**
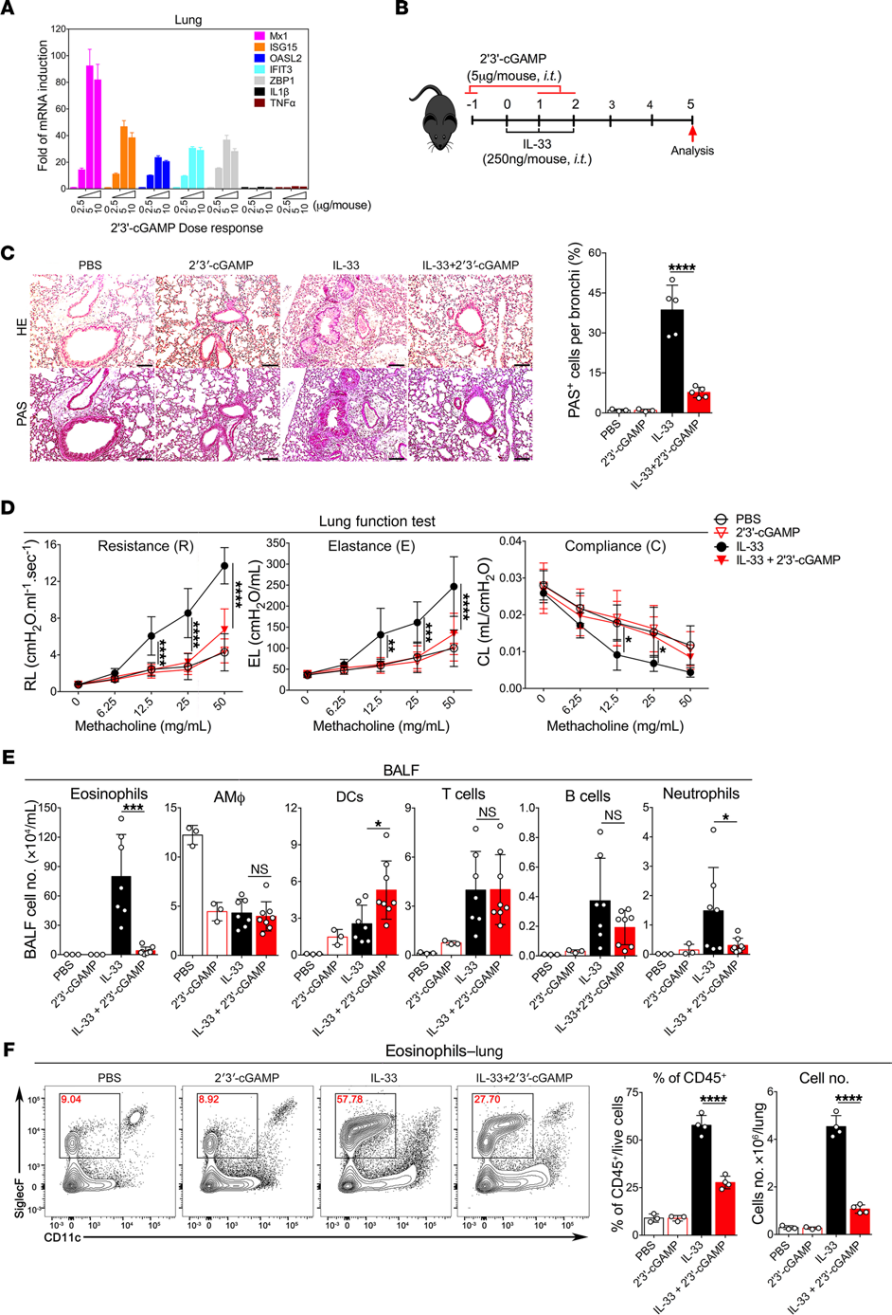
2′3′-cGAMP inhibits IL-33–induced type 2 lung inflammation. (**A**) Transcriptional induction of mouse lung gene expressions by 2′3′-cGAMP. Error bars represent standard error of triplicate assays. Representative data from 1 experiment are shown here. Similar results were obtained from at least 3 experiments. (**B**) Experimental setup illustrating the animal groups, regimen, and timeline. (**C**) Lung pathologies were assessed with H&E and PAS staining. Representative images (scale bars: 100 μm) and the percentage of PAS^+^ cells are shown here. Original magnification ×200 was used for counting the percentage of mucus-producing bronchial epithelial cells (-) (PAS^+^). (**D**) Lung functions were examined by Flexivent (Scireq). Airway resistance (R), elastance (E), and compliance (C) were measured after exposure to increasing doses (6.25–50 mg/mL) of aerosolized methacholine. A *P* value of less than 0.05 was considered significant, *n* = 4–6. Two-way ANOVA followed by Tukey’s multiple comparisons test was conducted. (**E**) Groups of mice as indicated were treated with PBS, 2′3′-cGAMP, IL-33, or IL-33 + 2′3′-cGAMP. Bronchoalveolar lavage fluid (BALF) was collected and analyzed for differential immune cell types. The result was a pool of 2 independent experiments. Open circles, *n* = 3–8 per group. A *P* value of less than 0.05 was considered significant using unpaired Student’s *t* test. (**F**) Administration of 2′3′-cGAMP decreased the percentage and number of lung eosinophils after exposure to IL-33. Open circles, *n* = 3–4 per group. A *P* value of less than 0.05 was considered significant using unpaired Student’s *t* test. *P < 0.05, **P < 0.01, ***P < 0.001, ****P < 0.0001.

It has been recently shown that 2′3′-cGAMP has a strong immune adjuvant effect to enhance both cellular and humoral immunity (19). However, it remains undetermined whether 2′3′-cGAMP is capable of modulating overzealous type 2 inflammatory responses in the context of eosinophilic asthma. To address this issue, we employed a mouse model of acute lung inflammation induced by a recombinant murine protein IL-33, which is known to robustly activate ILC2 cell–mediated lung inflammation (9, 24). We first determined whether 2′3′-cGAMP could initiate an immune response in mouse lungs. Mice were administered with increasing doses of 2′3′-cGAMP followed by examining lung gene expressions by real-time quantitative PCR (RT-qPCR). We found that multiple IFN-stimulated genes (ISGs) (Mx1, ISG15, IFIT3, and OASL2) were strongly induced to reach a peak level of expression by 2′3′-cGAMP at a dose of 5 μg/mouse. Notably, it appears that at this concentration of 2′3′-cGAMP, the induction of 2 proinflammatory genes IL-1β and TNF-α were not obvious (Figure 1A). Thus, we chose this dose for the subsequent in vivo experiments in combination with IL-33 (Figure 1B). We found that 2′3′-cGAMP treatment drastically improved IL-33–induced lung pathology characterized by less infiltration of inflammatory cells, reduced epithelial cell hyperplasia, and decreased percentage of mucus-producing (PAS^+^) epithelial cells (Figure 1C). Functionally, 2′3′-cGAMP treatment resulted in substantially reduced airway resistance and elastance, and increased compliance in response to methacholine (Figure 1D). Consistently, among all analyzed cell types, eosinophils in both bronchoalveolar lavage fluid (BALF) and lungs were dramatically reduced (Figure 1, E and F; the FACS gating strategy is shown in Supplemental Figure 1; supplemental material available online with this article; https://doi.org/10.1172/jci.insight.143509DS1; see complete unedited blots in the supplemental material). Notably, the number of neutrophils was also significantly reduced when compared with the IL-33–treated group. Taken together, these results suggest that 2′3′-cGAMP treatment triggered an immune response that strongly inhibits the development of IL-33–driven lung eosinophilia.

### 2′3′-cGAMP ameliorates lung eosinophilia and AHR induced by a fungal allergen, *A*. *flavus*.

**Figure 2:**
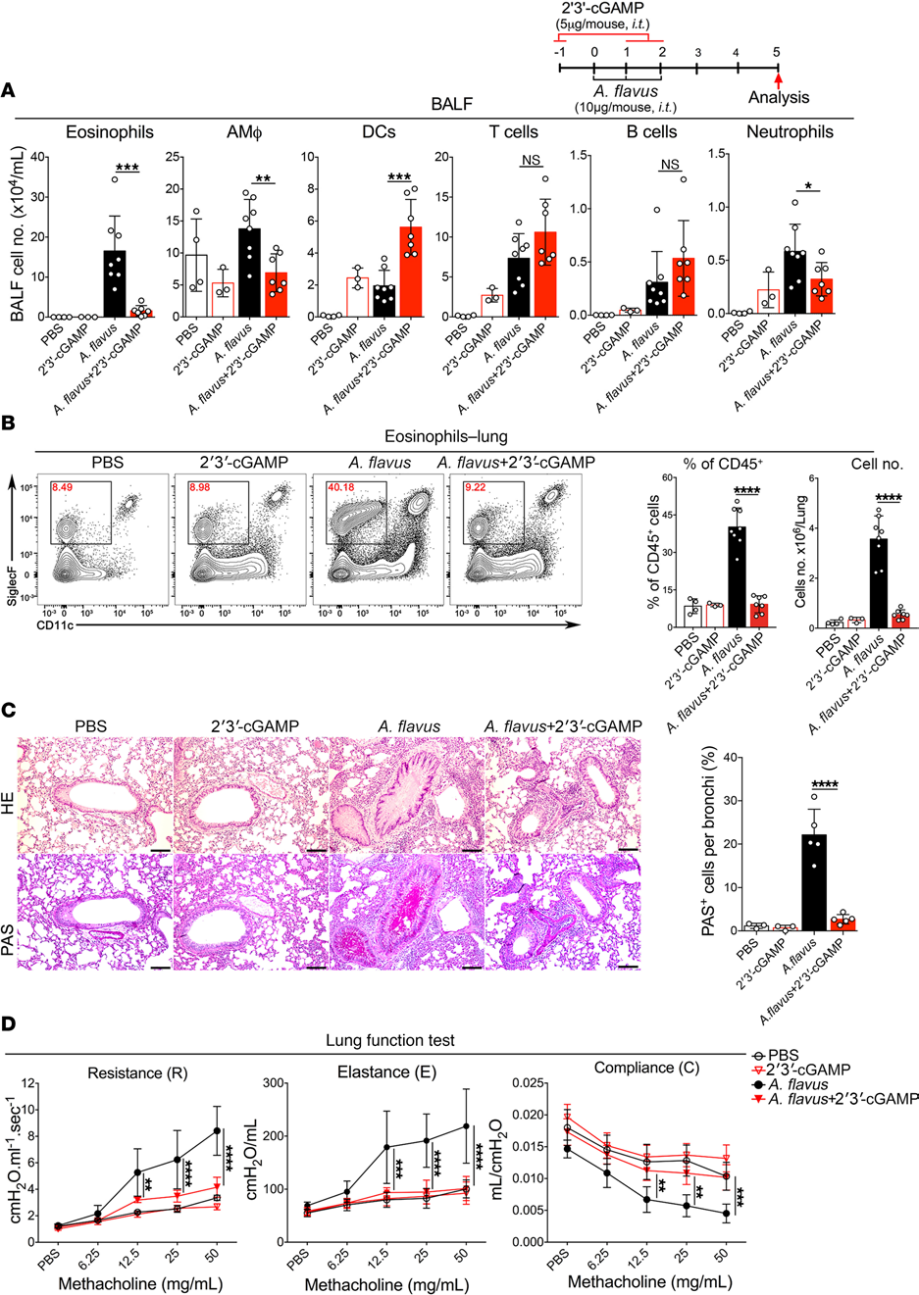
2′3′-cGAMP inhibits *A*. *flavus*-induced type 2 lung inflammation. (**A**) Groups of mice as indicated were treated with PBS, 2′3′-cGAMP, *A*. *flavus*, or *A*. *flavus* + 2′3′-cGAMP. BALF was collected and analyzed for differential immune cell types. The result was a pool of 2 independent experiments. Open circles, *n* = 3–7 per group. A *P* value of less than 0.05 was considered significant using unpaired Student’s *t* test. *P *<* 0.05, **P *<* 0.01, ***P *<* 0.001. (**B**) Administration of 2′3′-cGAMP decreased the percentage and number of lung eosinophils after exposure to *A*. *flavus*. Open circles, *n* = 3–8 per group. A *P* value of less than 0.05 was considered significant using unpaired Student’s *t* test. ****P < 0.0001. (**C**) Lung pathologies were assessed with H&E and PAS staining. Representative images (scale bars: 100 μm) and the percentage of PAS^+^ cells are shown here. Original magnification ×200 was used for counting the percentage of mucus-producing bronchial epithelial cells (-) (PAS^+^). ****P < 0.0001. (**D**) Airway hyperreactivity (AHR) was examined by Flexivent (Scireq). Airway resistance (R) was measured after exposure to increasing doses (6.25–50 mg/mL) of aerosolized methacholine. Open circles, *n* = 3–5 per group. A *P* value of less than 0.05 was considered significant. Statistics performed by 2-way ANOVA followed by Tukey’s multiple comparisons test. **P < 0.01, ***P < 0.001, ****P < 0.0001.

To evaluate the therapeutic benefit of 2′3′-cGAMP in the context of environmental allergen-driven type 2 lung inflammation, we used a clinically relevant model caused by a fungal allergen, *A*. *flavus*, that has been recently shown to act through the IL-33–mediated pathway (25). As with IL-33 described above, we challenged mice with 10 μg extract of *A*. *flavus* after exposing mice to 2′3′-cGAMP. We found that *A*. *flavus*–driven type 2 inflammatory markers were suppressed, as evidenced by the decrease in eosinophil infiltration of BALF and lung in 2′3′-cGAMP–treated mice (Figure 2, A and B). In addition, the number of neutrophils was also significantly reduced when compared with the *A*. *flavus*–treated group. 2′3′-cGAMP treatment drastically improved *A*. *flavus*–induced lung pathology characterized by infiltration of massive inflammatory cells, mucus overproduction (PAS^+^ epithelial cells) and epithelial cell hyperplasia (Figure 2C). Consistent with these observations, 2′3′-cGAMP treatment improved lung function as evidenced by reduced airway resistance, elastance, and improved compliance (Figure 2D). Thus, these data indicate that 2′3′-cGAMP treatment can activate a protective response to attenuate acute type 2 lung inflammation induced by a clinically relevant fungal allergen.

### 2′3′-cGAMP inhibits ILC2 cell–driven type 2 lung inflammation.

**Figure 3:**
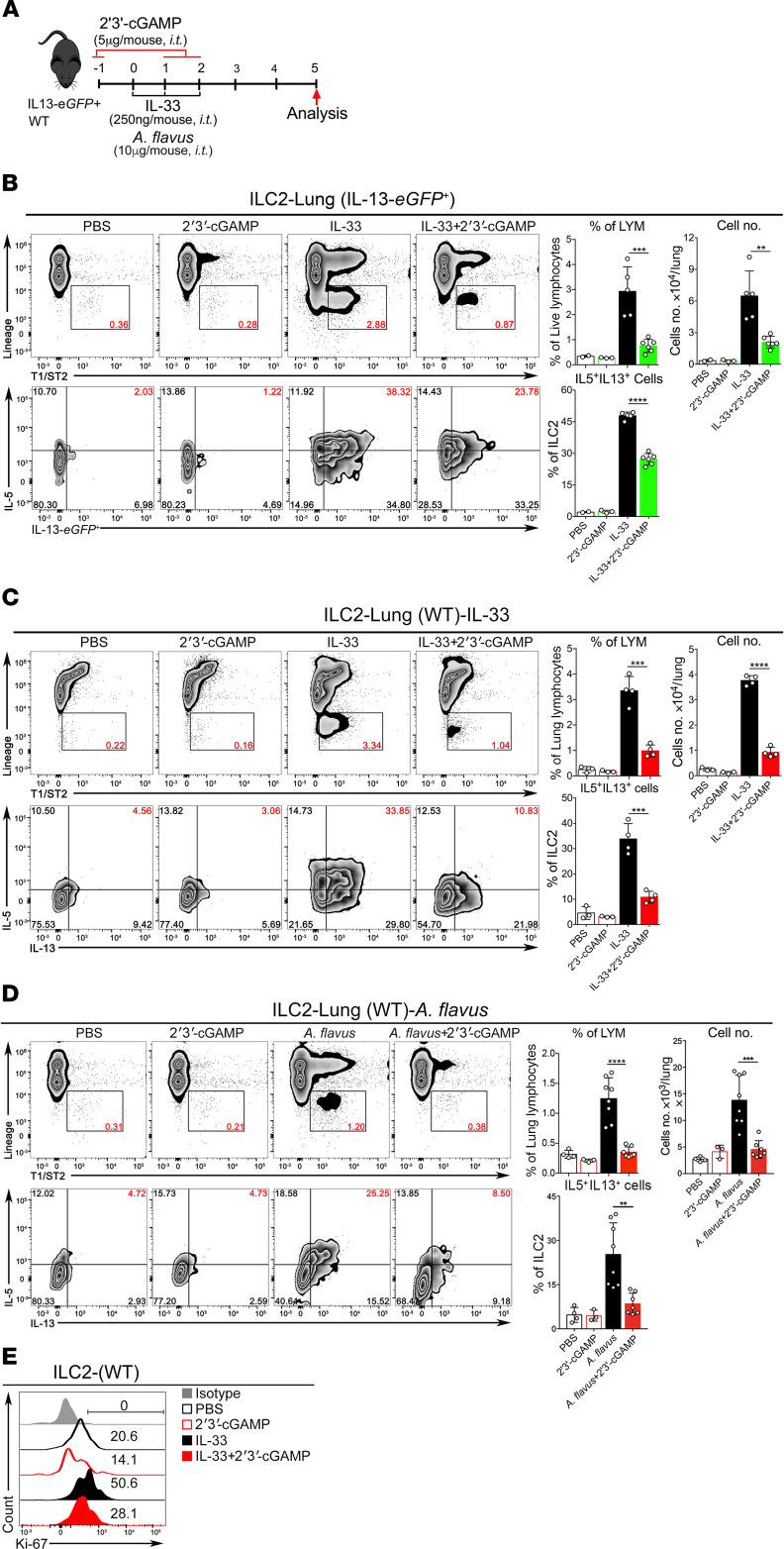
2′3′-cGAMP inhibits ILC2 cell activation and proliferation induced by IL-33 and *A*. *flavus* in WT or IL-13 reporter mice. (**A**) Experimental protocol showing the animal groups (IL-13-*eGFP*^+^ and WT), the corresponding treatment regimen and timeline. (**B**) The heterozygous IL-13-*eGFP*^+^ mice were treated with PBS, 2′3′-cGAMP, IL-33, or IL-33 + 2′3′-cGAMP. Lung single cell suspensions were prepared and the number of ILC2 cells in lungs were analyzed. In addition, lung cells were stimulated with phorbol 12-myristate 13-acetate in cultures as described in the Methods. The percentage of IL-5^+^IL-13^+^ double-positive ILC2 cells in lungs was analyzed (*n* = 2–6 per group as indicated with open circles). (**C**) Similar to **B**, instead, WT mice were used (*n* = 3–4 per group as indicated with open circles). (**D**) Similar to **C**, instead of IL-33, WT mice were treated with *A*. *flavus* (*n* = 3–8 per group as indicated with open circles). For **A**–**D**, a *P* value of less than 0.05 was considered significant using unpaired Student’s *t* test. **P < 0.01, ***P < 0.001, ****P < 0.0001. (**E**) WT mice were treated with PBS, 2′3′-cGAMP, IL-33, or IL-33 + 2′3′-cGAMP. The lung ILC2 cells were analyzed with Ki-67 staining and isotype antibody. The result is representative of 2 independent experiments.

**Figure 4:**
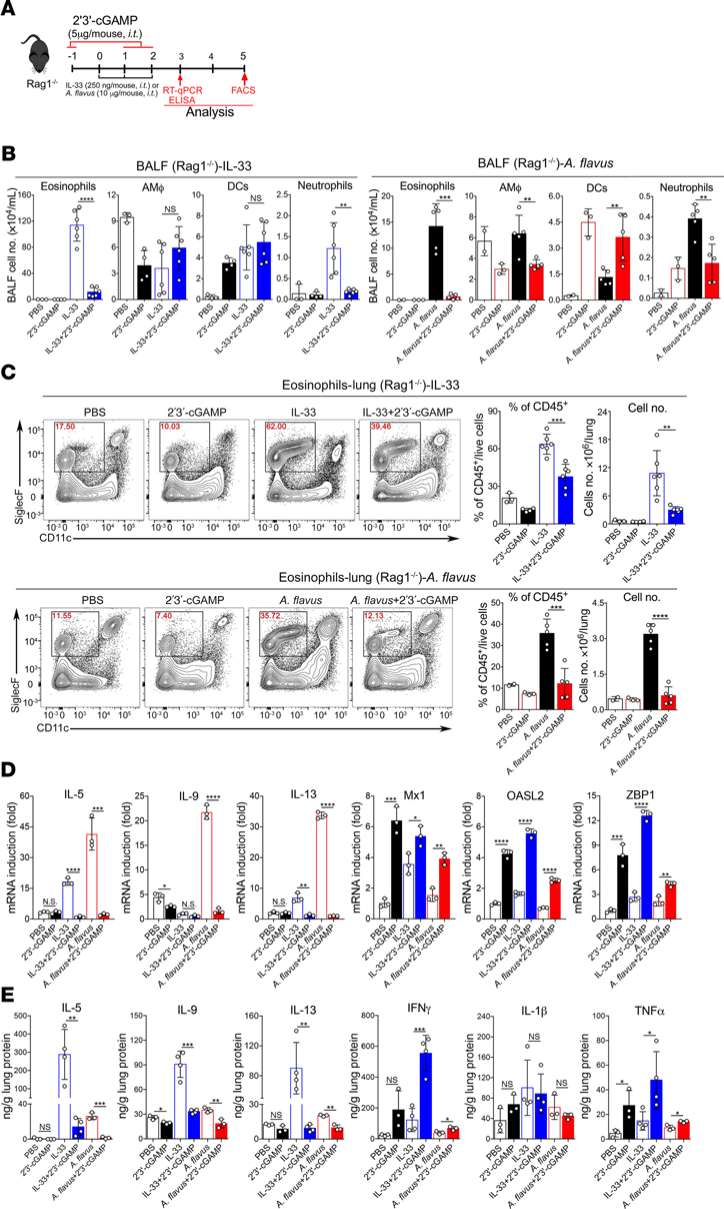
2′3′-cGAMP inhibits type 2 lung inflammation induced by IL-33 and *A*. *flavus* in Rag1^–/–^ mice. (**A**) Experimental protocol showing the animal groups of Rag1****^–/–^**** mice and the corresponding treatment regimen and timeline. (**B**) Administration of 2′3′-cGAMP into Rag1****^–/–^**** mice decreased the number of airway eosinophils. BALF analysis of Rag1****^–/–^**** mice were treated with 2 kinds of experimental regimens, PBS, 2′3′-cGAMP, IL-33, IL-33 +2 ′3′-cGAMP (left panel) or PBS, 2′3′-cGAMP, *A*. *flavus*, *A*. *flavus* + 2′3′-cGAMP (right panel). (**C**) Similar to **B**, administration of 2′3′-cGAMP into Rag1****^–/–^**** mice decreased the percentage and number of lung eosinophils after exposure to IL-33 (top panel) or *A*. *flavus* (bottom panel) (*n* = 2–5 per group as indicated with open circles, a *P* value of less than 0.05 was considered significant, unpaired Student’s *t* test. **P < 0.01, ***P < 0.001, ****P < 0.0001). (**D**) Similar to **B**, instead, lung samples were collected on day 3 for RNA extraction, then RT-qPCR analysis of the selected type 2 effector cytokines and IFN-stimulated genes (ISGs) as indicated were performed. Error bars represent standard error of triplicate assays. Representative data from 1 experiment are shown here. Similar results were obtained from at least 3 experiments. (**E**) Similar to **B**, instead, lung samples were collected and homogenized on day 3 for protein extractions, which were used for measuring the level of the selected cytokine as indicated by ELISA. For **D** and **E**, *n* = 3–4 per group as indicated with open circles. A *P* value of less than 0.05 was considered significant using unpaired Student’s *t* test. *P < 0.05, **P < 0.01, ***P < 0.001, ****P < 0.0001.

Next, we determined whether 2′3′-cGAMP could affect the function and proliferation of lung ILC2 cells in vivo activated by IL-33 or *A*. *flavus*. To easily track the activated population of ILC2 cells, we performed experiments with IL-13 reporter strain (the heterozygous mouse, IL-13-*eGFP^+^*, ref. 26) in addition to WT mice (Figure 3A). The gating strategy for lung ILC2 cells is shown in Supplemental Figure 2. ILC2 cell expansion was significantly suppressed, as determined by the reduction in total lung ILC2 cell numbers in both strains of mice in the context of IL-33 or *A*. *flavus* treatment. Functionally, the percentage of activated ILC2 cells (IL-5 and IL-13 double-positive) was also decreased upon treatment of 2′3′-cGAMP (Figure 3, B–D). Consistent with previous experiments, eosinophils in the BALF and lungs were significantly reduced in 2′3′-cGAMP–treated IL-13-*eGFP^+^* mice (Supplemental Figure 3). Further, 2′3′-cGAMP–mediated inhibitory effects on the ILC2 cell proliferation in WT mice was confirmed by Ki-67 staining (Figure 3E). To further rule out the involvement of adaptive immunity in 2′3′-cGAMP–induced suppressive effects, Rag1^–/–^ mice that lack mature B and T cells were tested in the context of IL-33 or *A*. *flavus* exposure (Figure 4A). FACS analysis revealed that upon exposures to either IL-33 or *A*. *flavus*, Rag1^–/–^ mice can develop severe type 2 lung inflammation characterized by the increased eosinophils in both BALF and lungs (Figure 4, B and C); elevated level of mRNA and protein expressions of type 2 effector cytokines such as IL-5, IL-9, and IL-13 (Figure 4, D and E); percentages of IL-5^+^ and IL-13^+^ double-positive cells; and total numbers of lung ILC2 cells (Supplemental Figure 4). Similar to the 2′3′-cGAMP–treated IL-13-*eGFP^+^* and WT mice, all above examined parameters of type 2 inflammation in Rag1^–/–^ mice were significantly reduced by 2′3′-cGAMP (Figure 4, B–E, and Supplemental Figure 4). Taken together, these results strongly suggest that 2′3′-cGAMP activates an innate immune signaling pathway to negatively regulate ILC2 cell–induced eosinophilic lung inflammation and that suppression is independent of the adaptive immunity in mice.

### 2′3′-cGAMP inhibits IL-33–, *A*. *flavus*–, or house dust mite extract–induced type 2 inflammation via STING/IFN-I signaling.

**Figure 5:**
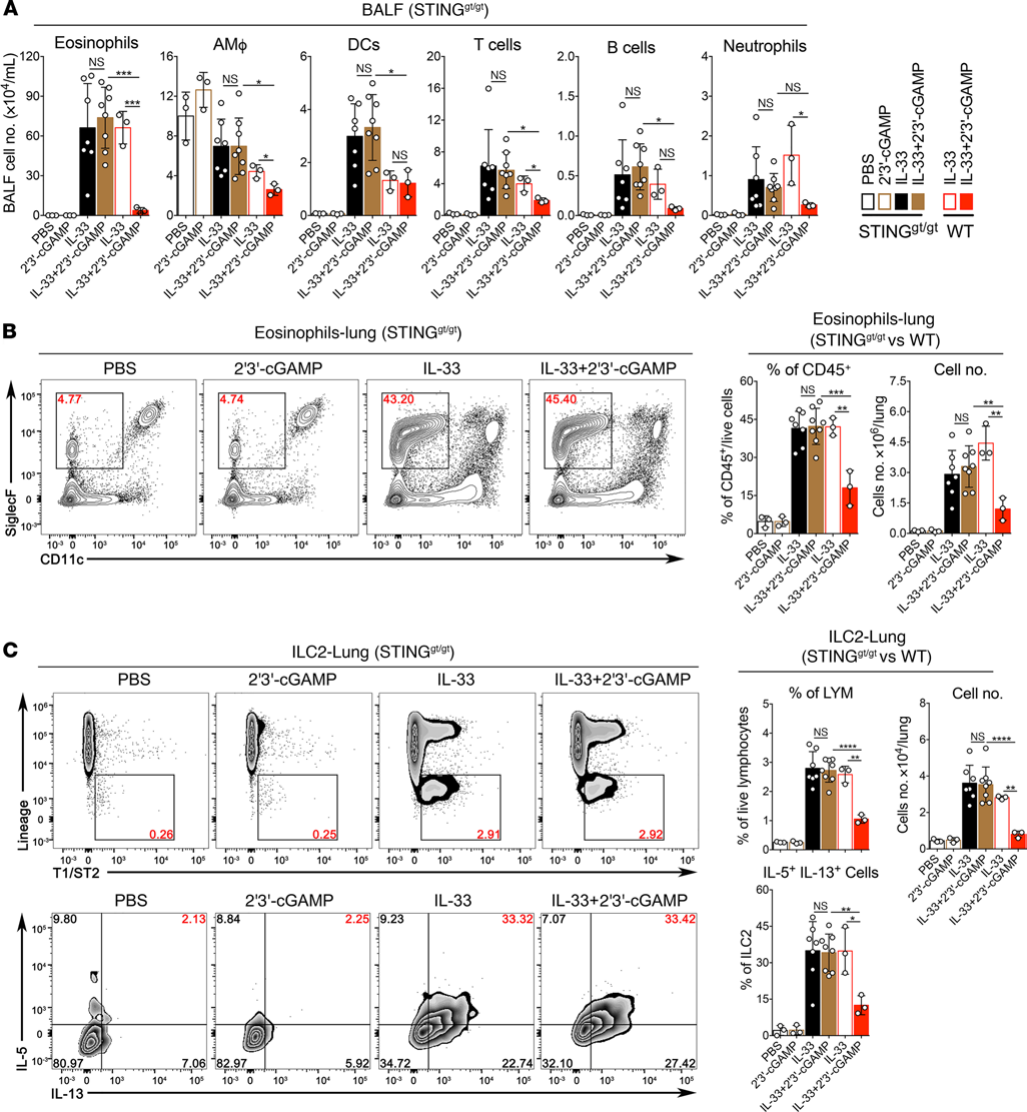
The inhibitory effect of 2′3′-cGAMP is abolished in STING-deficient mice. Groups of STING^gt/gt^ and WT mice were treated with PBS, 2′3′-cGAMP, IL-33, or IL-33 + 2′3′-cGAMP as indicated. BALF was collected and analyzed for differential immune cell types. (**A**) In contrast to WT mice, administration of 2′3′-cGAMP into STING^gt/gt^ mice did not significantly change number of airway eosinophils after exposure to IL-33. (**B**) Similar to **A**, administration of 2′3′-cGAMP in STING^gt/gt^ mice did not significantly change the percentage and number of lung eosinophils after exposure to IL-33. (**C**) Similar to **A**, the number and percentage of IL-5^+^IL-13^+^ double- positive ILC2 cells in lungs of STING^gt/gt^ mice were analyzed (*n* = 3–5 per group as indicated with open circles, a *P* value greater than or equal to 0.05 was not considered significant [NS], Student’s unpaired *t* test. *P < 0.05, **P < 0.01, ***P < 0.001, ****P < 0.0001).

**Figure 6:**
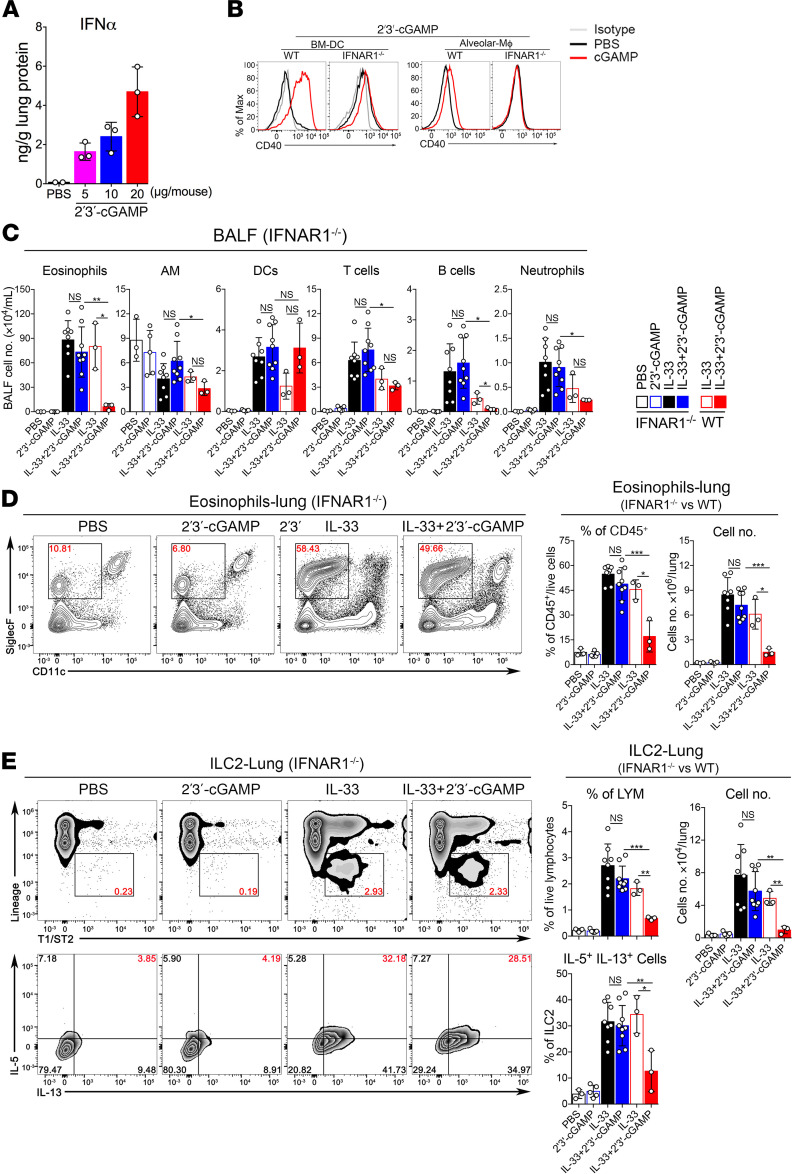
The inhibitory effect of 2′3′-cGAMP is mediated by the type I IFN signaling pathway. (**A**) Induction of IFN-α protein after administration of 2′3′-cGAMP at 5, 10, and 20 μg/mouse for 24 hours. The mouse lungs from WT mice were processed for the ELISA measurement. (**B**) BM-DCs and alveolar macrophages derived from WT and IFNAR1^–/–^ mice were treated with 2′3′-cGAMP. Then, the expression of a costimulatory molecule CD40 was analyzed by FACS. (**C**) Groups of IFNAR1^–/–^ and WT mice were treated with PBS, 2′3′-cGAMP, IL-33, or IL-33 + 2′3′-cGAMP as indicated. BALF was collected and analyzed for differential immune cell types. In contrast to WT mice, administration of 2′3′-cGAMP into IFNAR1^–/–^ mice did not significantly change number of airway eosinophils after exposure to IL-33. (**D**) Administration of 2′3′-cGAMP in IFNAR1^–/–^ mice did not significantly decrease the percentage and number of lung eosinophils after exposure to IL-33. (**E**) Similar to **C**, the number and percentage of IL-5^+^IL-13^+^ double-positive ILC2 cells in lungs of IFNAR1^–/–^ mice were analyzed (*n* = 3–7 per group as indicated with open circles, a *P* value of greater than or equal to 0.05 was not considered significant [NS], Student’s unpaired *t* test, *P < 0.05, **P < 0.01, ***P < 0.001).

Next, we investigated whether the inhibitory effect of 2′3′-cGAMP could be dependent on the STING/IFN-I signaling pathway. As expected, the effect of 2′3′-cGAMP on IL-33–induced type 2 lung inflammation was completely abolished in STING goldenticket/goldenticket (STING^gt/gt^) mice (Figure 5, A–C), indicating the in vivo specificity of 2′3′-cGAMP–activated innate immune responses. As responses are known to be a hallmark of the 2′3′-cGAMP/STING signaling (18–20), we then measured the level of IFN-α protein in mouse lungs by ELISA. 2′3′-cGAMP treatment, in a dose-dependent manner, triggered a robust production of IFN-α protein, which was detected from total lung homogenates (Figure 6A). It is very likely that activation of the 2′3′-cGAMP/STING pathway would generate multiple effector molecules in mouse lungs besides IFN-I. To demonstrate a possible role of IFN-I signaling, we performed a series of experiments using IFNAR1-deficient mice. The expression of a stimulatory molecule CD40 in bone marrow–derived DCs (BM-DCs) and alveolar macrophages derived from IFNAR1^–/–^ mice were greatly reduced when stimulated with 2′3′-cGAMP (Figure 6B). More importantly, in contrast to WT mice shown in Figure 1 and Figure 2, IFNAR1^–/–^ mice treated with either IL-33 or IL-33 + 2′3′-cGAMP did not show any significant differences in eosinophils and ILC2 cells (number and percentage) in BALF or lungs (Figure 6, C–E). We also explored the possibility whether the eosinophilia and activation of ILC2 cells could be inhibited by 2′3′-cGAMP codelivered or delivered afterward with *A*. *flavus* in Rag1^–/–^ mice (Supplemental Figure 5) or WT mice (Supplemental Figure 6). In addition, we examined whether 2′3′-cGAMP treatment can affect the established type 2 lung inflammation induced by house dust mite extract (HDM), a more physiologically relevant aeroallergen (Supplemental Figure 7A). As shown in Supplemental Figure 7, B and D, 2′3′-cGAMP treatment significantly inhibited HDM-induced lung eosinophilia and activation of ILC2 cells. In all of these treatments, 2′3′-cGAMP was effective in suppressing type 2 immunopathology. Taken together, these results suggest that the STING/IFN-I signaling axis mediates the effector function of 2′3′-cGAMP in vivo to negatively regulate type 2 lung inflammation induced by IL-33 and natural allergens likely via suppressing the activation of ILC2 cells.

### Activation of alveolar macrophages by 2′3′-cGAMP leads to the production of ILC2 cell inhibitory factors.

**Figure 7:**
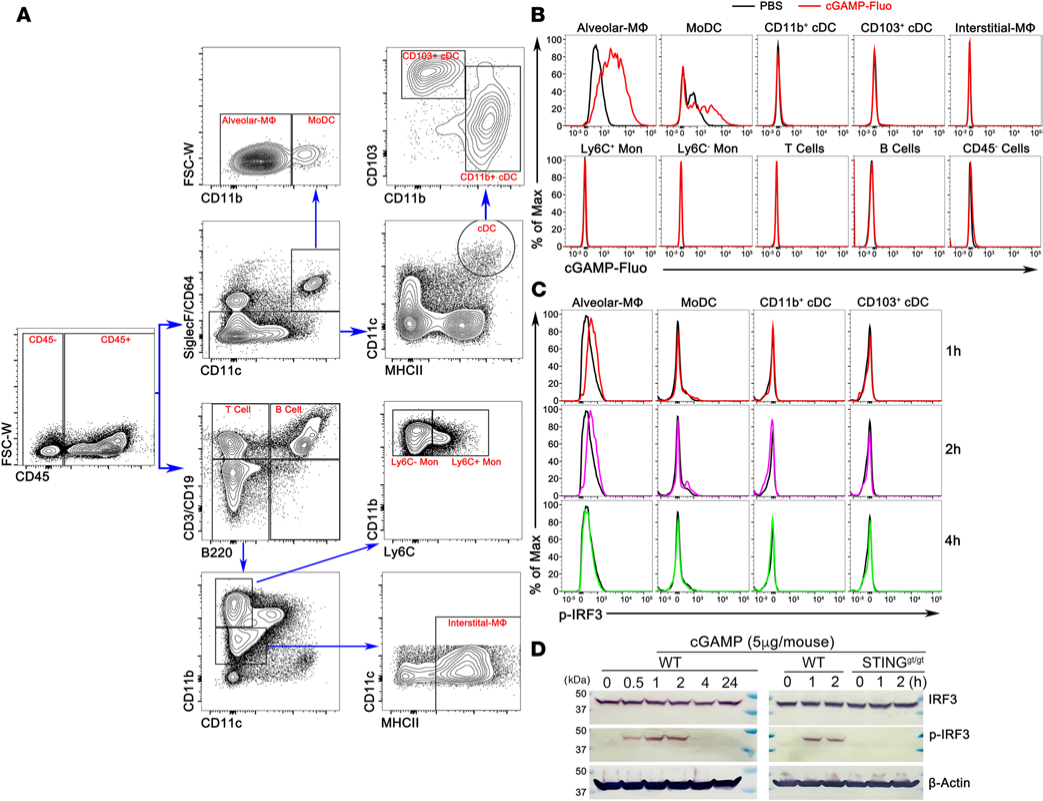
2′3′-cGAMP is mainly taken up by alveolar macrophages in vivo. (**A**) FACS gating strategy for identification of specific lung cell types by staining cell surface markers as indicated in the Methods. (**B**) The fluorescence-labeled 2′3′-cGAMP was mainly detected by FACS analysis in alveolar macrophages, and to a lesser extent, monocyte-derived DCs (MoDCs), but not in other lung cell types. The result is representative of 3 independent experiments. (**C**) FACS analysis shows that the phosphorylated form of IRF3 can be detected in alveolar macrophages at 1–2 hours after the 2′3′-cGAMP treatment. The result is representative of 3 independent experiments. (**D**) Western blot analysis of homogenates of mouse lungs shows that 2′3′-cGAMP treatment rapidly activates the STING-mediated pathway within 1–2 hours that results in the phosphorylation of IRF3.

**Figure 8:**
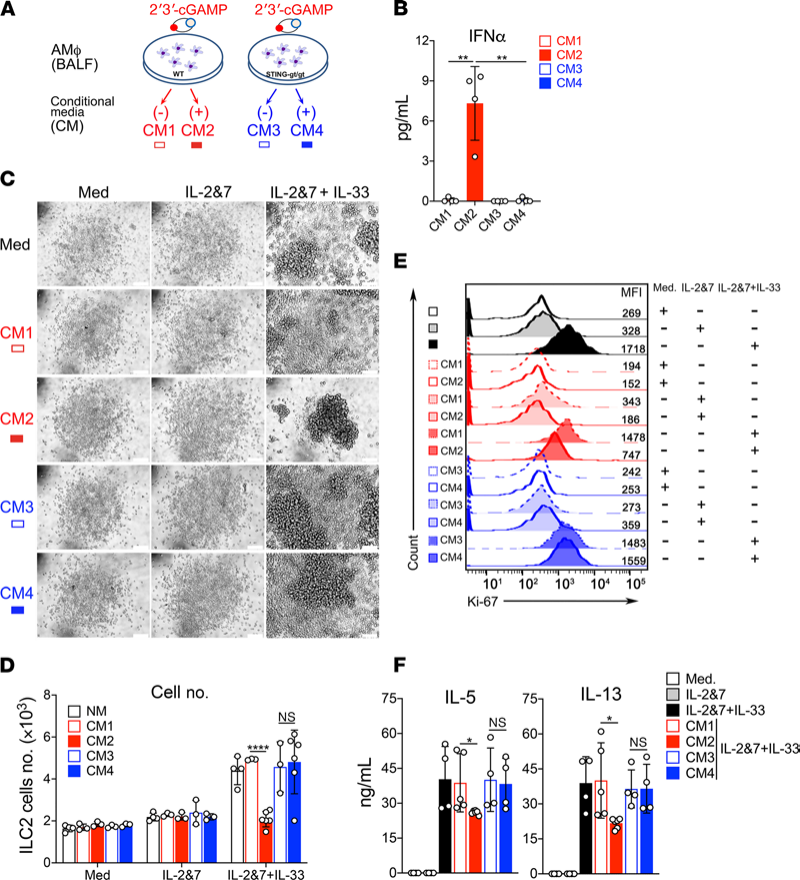
2′3′-cGAMP stimulates alveolar macrophages to produce ILC2-inhibitory factors. (**A**) Diagram showing the generation of conditioned media (CM) from the ex vivo cultured alveolar macrophage (AMΦ) cells from WT or STING^gt/gt^ mice that were stimulated with or without 2′3′-cGAMP. (**B**) The level of IFN-α in the CM was measured by ELISA. (**C**) Light microscopic images showing the growth of mouse ILC2 cells in the presence of the corresponding CM as indicated. (**D**) FACS showing the number of murine ILC2 cells in the presence of the CM. (**E**) Ki-67 staining of mouse ILC2 cells in the presence of the CM. (**F**) ELISA measuring the production of IL-5 and IL-13 by mouse ILC2 cells in the presence of the CM. Statistics for **D** were performed using 2-way ANOVA followed by Tukey’s multiple comparisons test by comparing mouse ILC2 cells treated with CM as indicated. A *P* value greater than or equal to 0.05 was not considered significant (NS). *P < 0.05, **P < 0.01, ****P < 0.0001.

To identify lung cell types that are responsible for taking up 2′3′-cGAMP, we performed a flow cytometric analysis of lung cells (FACS gating strategy, Figure 7A). Mice were treated with fluorescently labeled 2′3′-cGAMP (cGAMP-Fluo), and lungs were collected after 16 hours. Lung tissues were then digested, and cells were stained with various cell type–specific surface makers. Interestingly, cGAMP-Fluo was mainly detected in alveolar macrophages and, to a lesser extent, monocyte-derived DCs (MoDCs), but not in other examined lung immune cells such as conventional DCs, interstitial macrophages, monocytes, T cells, B cells, and nonimmune cells (CD45^–^ cells, Figure 7B). FACS analysis further revealed that the phosphorylated form of IFN regulatory factor 3 (IRF3) in alveolar macrophages appeared within 1–2 hours after the 2′3′-cGAMP treatment (Figure 7C). STING is indispensable for the in vivo activity of 2′3′-cGAMP because the phosphorylated form of IRF3 was only detected in the lung homogenates of WT, but not in STING^gt/gt^ mice (Figure 7D). To determine whether 2′3′-cGAMP–stimulated alveolar macrophages could produce IFN or ILC2 cell regulatory factors, we collected conditioned media from the cultured alveolar macrophages as illustrated in Figure 8A. As expected, IFN-α was only detected in the conditioned media (CM) of alveolar macrophages from WT, but not STING deficient mice by ELISA (Figure 8B). Moreover, although it had no effects on the nonactivated ILC2 cells under the conditions of media or IL-2 + IL-7, the CM from 2′3′-cGAMP–treated WT alveolar macrophages significantly impaired the growth and function of IL-33–activated ILC2 cells measured by the cell number and proliferation marker Ki-67 (Figure 8, C–E) and the cytokine levels of IL-5 and IL-13 (Figure 8F). To further demonstrate an important role of alveolar macrophages in mediating the effects of 2′3′-cGAMP in vivo, we have performed IL-33 treatments in 2 mouse lines lacking alveolar macrophages (Supplemental Figures 8A and 9A), Csf2^–/–^ and clodronate liposome-treated WT mice, in which type 2 inflammatory responses were poorly activated (Supplemental Figures 8 and 9). Nonetheless, it appears that the 2′3′-cGAMP administration could further reduce the numbers of eosinophils and ILC2 cells when compared with the mouse lines lacking alveolar macrophages treated with the IL-33 alone, implying that 2′3′-cGAMP might act on other cell types such as ILC2 in vivo. Overall, these results indicate that alveolar macrophages have the ability to take up extracellular 2′3′-cGAMP and turn on the STING/IRF3 pathway that may lead to the production of ILC2 inhibitory factors such as IFN-I.

### 2′3′-cGAMP directly suppresses the proliferation and cytokine production of human and mouse ILC2 cells in vitro. 

**Figure 9:**
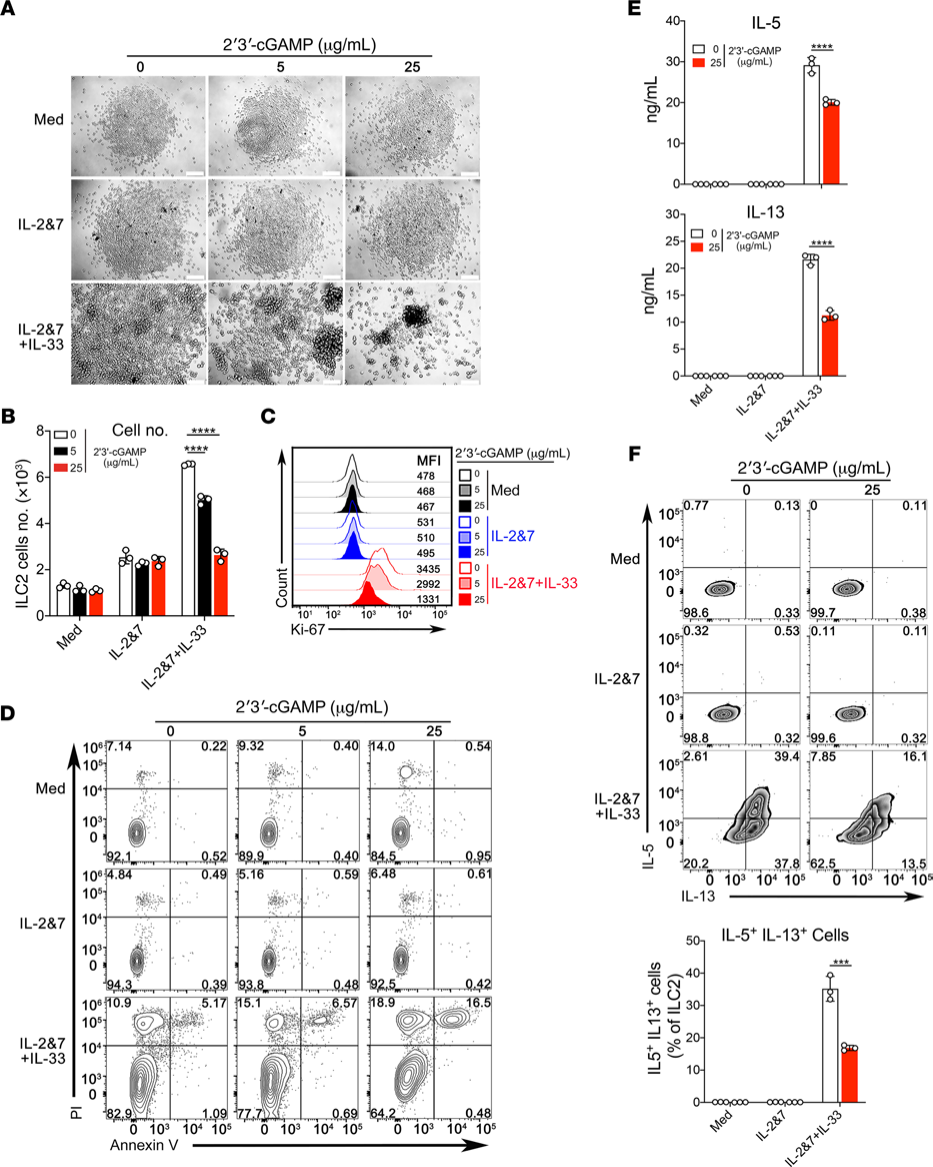
2′3′-cGAMP directly suppresses the proliferation and cytokine production of mouse ILC2 cells. (**A**) Light microscopic images showing the growth of mouse ILC2 cells in the presence of the increased concentration of 2′3′-cGAMP. (**B**) FACS showing the number of murine ILC2 cells in the presence of the increased concentration of 2′3′-cGAMP. (**C**) Ki-67 staining of murine ILC2 cells in the presence of the increased concentration of 2′3′-cGAMP. (**D**) Cell death of mouse ILC2 cells in the presence of the increased concentration of 2′3′-cGAMP. (**E**) ELISA measuring the production of IL-5 and IL-13 by mouse ILC2 cells in the presence of the increased concentration of 2′3′-cGAMP. (**F**) Intracellular staining of IL-5 and IL-13 in mouse ILC2 cells treated with 2′3′-cGAMP (the percentage of the double-positive cells was quantified, lower). Statistics were performed using 2-way ANOVA followed by Tukey’s multiple comparisons test by comparing media-treated mouse ILC2 cells with the individual treatment as indicated. A *P* value greater than or equal to 0.05 was not considered significant. ***P < 0.001, ****P < 0.0001.

**Figure 10:**
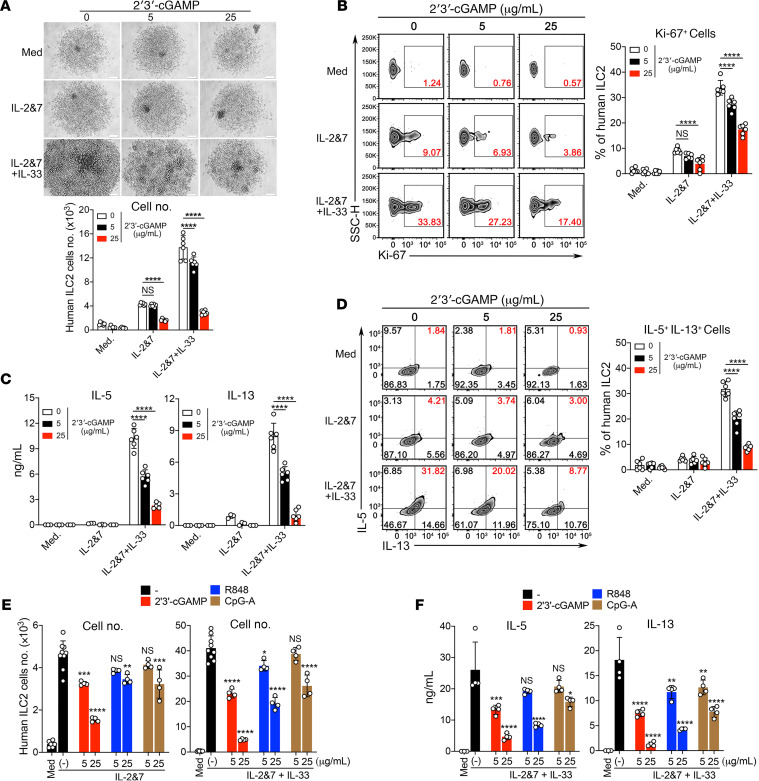
2′3′-cGAMP directly suppresses the proliferation and cytokine production of human ILC2 cells. (**A**) Light microscopic images and FACS showing the growth and number of human ILC2 cells in the presence of the increased concentration of 2′3′-cGAMP. (**B**) Ki-67 staining of human ILC2 cells in the presence of the increased concentration of 2′3′-cGAMP. (**C**) ELISA measuring the production of IL-5 and IL-13 by human ILC2 cells in the presence of the increased concentration of 2′3′-cGAMP. (**D**) Intracellular staining of IL-5 and IL-13 in human ILC2 cells treated with the increased concentration of 2′3′-cGAMP (the quantification of the percentage of the double-positive cells, right). (**E**) Human ILC2 cells cultured under either IL-2&7 or IL-2&7 + IL-33 were treated with 2′3′-cGAMP, R848, or CpG-A as indicated. FACS showing the growth of human ILC2 cells. (**F**) Same as **E**. The production of IL-5 and IL-3 was measured by ELISA. The result is representative of 3 independent experiments. Statistics were performed using 2-way ANOVA followed by Tukey’s multiple comparisons test by comparing media or mock (-)-treated human ILC2 cells to the individual treatment as indicated. A *P* value of greater than or equal to 0.05 was not considered significant (NS). *P < 0.05, **P < 0.01, ***P < 0.001, ****P < 0.0001.

**Figure 11:**
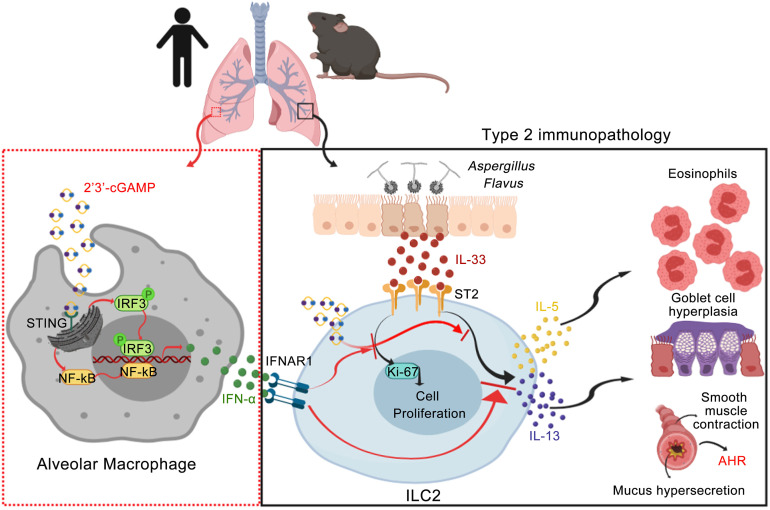
A proposed working model illustrates the potential mechanism through which 2′3′-cGAMP inhibits type 2 immunopathology by targeting human and mouse alveolar macrophages and ILC2 cells. Artwork was initially created in BioRender, https://app.biorender.com.

Because we have shown above that 2′3′-cGAMP treatment could act on alveolar macrophages to negatively regulate ILC2 cell–driven type 2 inflammation, next we wanted to assess its potential effect on ILC2 cells in vitro. The purified mouse and human ILC2 cells (Supplemental Figure 10) were cultured under conditions of media alone, IL-2 + IL-7, or IL-2 + IL-7 + IL-33 and stimulated with 2′3′-cGAMP. Indeed, the purified mouse and human ILC2 cells were able to take up 2′3′-cGAMP and activate the STING/IFN-I pathway (Supplemental Figure 11). Further, in a concentration-dependent manner, 2′3′-cGAMP strongly suppressed the proliferation of both mouse and human ILC2 cells demonstrated by the cell density, the cell number, and the level of Ki-67 (Figure 9, A–C; and Figure 10, A and B). We found that this suppression by 2′3′-cGAMP was partly attributed to the induction of cell death (Figure 9D). Moreover, 2′3′-cGAMP strongly suppressed the production of IL-5 and IL-13 by mouse and human ILC2 cells activated by IL-33 (Figure 9E and Figure 10C). Intracellular cytokine staining also demonstrated that 2′3′-cGAMP also suppressed the cytokine expression inside the ILC2 cells (Figure 9F and Figure 10D). Moreover, 2′3′-cGAMP at a lower concentration (5 μg/mL), when compared with 2 other TLR agonists (R848, a TLR7 agonist, and CpG-A, a TLR9 agonist), appears to be more potent in directly suppressing both the growth and production of type 2 effector cytokines of human ILC2 cells (Figure 10, E and F). Notably, at a higher concentration (25 μg/mL) all 3 agents had inhibitory effects on human ILC2 cells. Collectively, these results indicate that 2′3′-cGAMP can directly suppress the activation of ILC2 cell function induced by IL-33. Taken together, our data indicate that IFN-I signaling mediates the effector function of 2′3′-cGAMP to negatively regulate allergen-induced type 2 immune responses. Based on these findings, as depicted in Figure 11, we propose a working model for 2′3′-cGAMP in modulating ILC2 cell–mediated type 2 immunity. After the respiratory delivery, 2′3′-cGAMP is first taken up by alveolar macrophages, where it activates the STING-mediated pathway to induce the production of IFN-I, which may in turn act on ILC2 cells to restrain their abilities to proliferate and produce type 2 cytokines. In addition, 2′3′-cGAMP seems to be able to directly suppress the function of activated ILC2 cells in the context of exposure to IL-33 and environmental allergens such as *A*. *flavus*.

## Discussion

In this study, we reveal an important role for 2′3′-cGAMP in negatively regulating type 2 inflammation induced by IL-33, a fungal allergen, and HDM. We demonstrate that 2′3′-cGAMP administration efficiently protects mice from both IL-33– and a fungal allergen-induced AHR. Mechanistically, 2′3′-cGAMP can directly act on both alveolar macrophages and ILC2 cells. In alveolar macrophages, 2′3′-cGAMP activates the STING/IRF3 pathway that leads to a rapid production of ILC2 cell suppressive factors such as IFN-α. These data provide proof-of-concept evidence to support a therapeutic value of 2′3′-cGAMP in preventing and mitigating lung inflammation of eosinophilic asthma.

A few recent studies have shown that IFN-I directly inhibits the function and proliferation of ILC2 cells in vitro and in vivo during influenza A infection or treatment with TLR agonists such as CpG-DNA and R848 (15, 16, 27–30). Interestingly, our results suggest that the inhibitory effects of 2′3′-cGAMP are likely to be attributed, at least in part, to its ability in triggering production of IFN in mouse lungs, which generates a Th1-dominant cytokine milieu that is suppressive to ILC2 cells, which may further lead to a reduced level of Th2-biased adaptive immune response. In a recent phase II clinical trial, administering inhaled recombinant IFN-α/β was effective in treating steroid-resistant eosinophilic asthma (31). However, an IFN-based therapy is often associated with a wide array of adverse effects (32, 33). Therefore, instead of using cytokines directly, agonists that specifically stimulate the innate immune system are becoming more favorable therapeutics largely attributed to their lower toxicity and better immune response profile. In this regard, our study presents an important proof of principle for treating eosinophilic asthma through harnessing 2′3′-cGAMP-STING–mediated innate immune response.

Because of its inherent dual negative charges and the presence of an extracellular enzyme such as ectonucleotide pyrophosphatase/phosphodiesterase (34), an efficient in vivo delivery of 2′3′-cGAMP was usually considered to be very challenging without proper formulations. However, our results show that when administered intratracheally at a low dose, 2′3′-cGAMP was capable of inducing innate immune responses comprising IFN-I that were sufficient to inhibit ILC2 cell activation by IL-33 or a fungal allergen, *A*. *flavus*. Given that alveolar macrophages are the first line of phagocytic defense in lower airways and are approximately 50 times more numerous than conventional DCs, it was not unexpected that 2′3′-cGAMP was found to be mainly taken up by alveolar macrophages, and to a lesser extent, MoDCs in mouse lungs. It is possible that the 2′3′-cGAMP–activated alveolar macrophages can produce chemokines and cytokines, including IFN-I, to create a suppressive local environment against the development of overzealous type 2 inflammation upon exposures to various aeroallergens. In the context of allergic inflammation, inflamed lung tissue may become leaky and allow immune cells or cytokines to travel or penetrate across tissue barriers to act on ILC2 cells. However, the molecular mechanism of how 2′3′-cGAMP enters the cytosol of alveolar macrophages where it engages the downstream STING/IFN-I signaling pathway remains unknown. In this regard, 2 recent reports suggest that 2 proteins, cGAS and human SLC19A1, might be involved in trafficking of extracellular 2′3′-cGAMP into the cytoplasm of cultured macrophages and monocytes (35, 36). Additionally, in contrast to bacterial cyclic dinucleotides (3′3′-c-di-GMP, 3′3′-c-di-AMP, and 3′3′-cGAMP), which preferentially bind with high affinity and activate 2 new sensors ERAdP (37) and RECON (37–40), 2′3′-cGAMP is a much more potent and specific ligand for human STING (41, 42). Collectively, it appears that 2′3′-cGAMP would be a promising candidate for harnessing innate immunity to treat or prevent many human diseases, such as cancer and eosinophilic asthma.

At present, a number of STING agonists have entered clinical trials for cancer immunotherapy (21–23). It can also be envisioned that many issues or concerns such as adverse effects may arise and must be further addressed with independent experimental approaches. At the same time, there will be more exciting opportunities on the parallel development of these reagents for other applications such as treating eosinophilic diseases. Supported by this work, the use of 2′3′-cGAMP or other STING agonists as immune modulators to enhance the efficacy of merits future investigation.

To some extent, our current findings on 2′3′-cGAMP are consistent with a new report showing that a bacterial cyclic dinucleotide, c-di-GMP, can effectively inhibit IL-33– or *Alternaria*-induced type 2 inflammation in mice (43). However, it has also recently been reported that in another model of allergic inflammation, the administration of 2′3′-cGAMP had an adjuvant effect that exacerbated the HDM-induced Th2 response (44). This result is contrast to our data presented in Supplemental Figure 7, in which HDM-induced type 2 inflammation was attenuated by 2′3′-cGAMP administration. The exact causes for these discrepancies are unclear and could be ascribed to many things, such as the dose, timing, and delivery route for 2′3′-cGAMP. More preclinical experiments, however, are needed to resolve this controversy.

In conclusion, our results highlight the role of an allergen-independent, innate immune-driven effector function triggered by the 2′3′-cGAMP/STING/IFN-I signaling pathway, which robustly counteracts the rapidly activated ILC2 cells in the context of IL-33 and a fungal allergen-induced acute type 2 inflammation. From the therapeutic standpoint, our study suggests that further development of a formulated 2′3′-cGAMP for local delivery into the lungs may serve as an alternative approach for preventing or treating eosinophilic asthma.

## Methods

### Mice.

IL-13-*eGFP* reporter strain (26) and STING^gt/gt^ (45) mice have been described previously. WT C57BL/6J, Csf2^–/–^, IFNAR1^–/–^, and Rag1^–/–^ mice were purchased from the Jackson Laboratory. The IL-13-*eGFP*^+^ heterozygous mice were generated by intercrossing with WT C57BL/6J on campus. Mice were bred and maintained under specific pathogen–free conditions in the animal facility. Age-matched (8–10 weeks old) female mice were used for the experiments.

### Cells and reagents.

Bone marrow cells were collected from femurs and tibiae of mice. To obtain BM-DCs, about 10 million bone marrow cells were cultured in DMEM containing 10% FCS, antibiotics, and Flt3 ligand. After 7 days, mature DCs were harvested and cultured in 96-well plates for experiments. Media were changed every other day. 2′3′-cGAMP was purchased from InvivoGen.

### ELISA to detect cytokines in mouse lungs and cell culture supernatants.

For measuring cytokines in mouse lungs after 2′3′-cGAMP stimulation, the harvested lungs were washed once with cold PBS, transferred into 2 mL tubes, rapidly frozen into liquid-N2, and stored at −80°C. Later, to prepare lung homogenates, 1 mL tissue protein extraction reagent (T-PER) (Thermo Fisher Scientific) containing protease inhibitors (Roche) was added and homogenized by a BeadBeater (BioSpec). The lysates were transferred to a 1.5 mL tube and spun at 14,000*g* for 30 minutes at 4°C. Supernatant was collected for the ELISA measurement of cytokines. IFN-α in supernatant of alveolar macrophages culture and lung homogenates were measured with the ELISA kit (PBL Assay Science). Cytokines such as TNF-α, IL-5, IL-9, IL-13, and IFN-γ in lung homogenates and type 2 cytokines (IL-5 and IL-13) in supernatants of mouse or human ILC2 cell cultures were analyzed with ELISA kit (all purchased from Invitrogen), and IL-1β in lung homogenates was detected by the ELISA kit (R&D Systems). All final reactions were developed with TMB substrate (Thermo Fisher Scientific) and stopped by sulfuric acid (0.16 M), and the OD at 450 nm was measured.

### Western blot.

Western blot analysis was performed as previously described with some modifications (46). Briefly, protein extracts were obtained from the lungs of 8-week-old female mice treated with 2′3′-cGAMP (5 μg/mouse) for different time periods. One-half of the lung tissue was put into lysing Matrix D tube (MP Biomedicals) and immediately frozen in liquid N2. A total of 1.0 mL extraction buffer (8 M urea, 1% SDS, 0.15 M Tris-HCl, pH 7.5) was added, and samples were homogenized by a BeadBeater (BioSpec). The lysates were transferred to a 1.5 mL tube and spun at 14,000*g* for 30 minutes at 4°C. Supernatant was collected, and protein concentration was measured by Bradford protein assay (Pierce). A total of 55 μg of each sample was loaded and separated in 10% SDS/PAGE and transferred to PVDF (MilliporeSigma). Membranes were blocked with 5% nonfat milk and incubated with rabbit mAbs against IRF3, p-IRF3, and β-actin (Cell Signaling Technology).

### RT-qPCR.

Reverse transcription and RT-qPCR reactions were carried out using iScript cDNA synthesis kit and iQ SYBR Green Supermix (Bio-Rad). qPCR was performed on a Bio-Rad CFX384 Touch Real-Time PCR Detection System using the following primers: mouse primers, forward (5′→3′); reverse (5′→3′): Rpl19 (AAATCGCCAATGCCAACTC; TCTTCCCTATGCCCATATGC), IL-1β (TCTATACCTGTCCTGTGTAATG; GCTTGTGCTCTGCTTGTG), IFIT3 (TGGCCTACATAAAGCACCTAGATGG; CGCAAACTTTTGGCAAACTTGTCT), ISG15 (GAGCTAGAGCCTGCAGCAAT; TTCTGGGCAATCTGCTTCTT), Mx1 (TCTGAGGAGAGCCAGACGAT; ACTCTGGTCCCCAATGACAG), OASL2 (GGATGCCTGGGAGAGAATCG; TCGCCTGCTCTTCGAAACTG), TNF-α (CCTCCCTCTCATCAGTTCTATGG; GGCTACAGGCTTGTCACTCG), IL-5 (AGGATGCTTCTGCACTTGAG; CCTCATCGTCTCATTGCTTG), IL-9 (GAACATCACGTGTCCGTCCT; CGGCTTTTCTGCCTTTGCAT), IL-13 (TGAGCAACATCACACAAGACC; AGGCCATGCAATATCCTCTG).

### In vivo administration and FACS analysis of BALF and lung.

Mice were anesthetized by isoﬂurane inhalation, followed by 3 times of intratracheal administration with 2′3′-cGAMP (5 μg), rIL-33 (0.25 μg), or *A*. *flavus* protease allergen (10 μg) in 80 μL PBS as shown in Figure 1B. Mice were sacrificed at indicated times, and the trachea was catheterized and flushed with 1 mL ice-cold PBS-EDTA 3 times. Differential cells in BALF were labeled with antibodies as indicated, then mixed with counting beads (Spherotech) for further FACS analysis on a BD Celesta cell analyzer. Flow cytometry data were analyzed using FlowJo software. The antibodies and reagents for FACS analysis are as follows: SPHERO AccuCount Fluorescent (Spherotech, ACFP-70-5), Anti-Mouse Siglec-F PE (clone E50-2440) (BD Biosciences, 552126), Anti-Mouse CD19 Alexa Fluor 647 (clone 1D3) (BD Biosciences, 557684), Anti-Mouse CD3ε APC (clone 145-2C11) (BioLegend, 100322), Anti-Mouse MHC II APC-Cy7 (clone M5/114.15.2) (BioLegend, 10627), Anti-Mouse CD11c PE-Cy7 (clone N418) (Tonbo Biosciences, 60-0114-U100), Anti-Mouse CD11b V450 Rat (clone M1/70) (BD Biosciences, 560456), Anti-Mouse Ly-6G FITC (clone RB6-8C5) (Invitrogen, 11-5931-82), Anti-Mouse Fixable Viability Dye eFluor 506 (Invitrogen, 65-0866-14), Anti-Mouse CD45 PerCP-Cy5.5 (clone 30-F11) (BioLegend, 103130), Anti-Mouse CD45 APC-Cy7 (clone 30-F11) (BD Biosciences, 561037), Anti-Mouse CD103 Alexa Fluor 647 (clone 2ET) (BioLegend, 121410), Anti-Mouse CD64 PE (clone X54-5/7.1) (BioLegend, 139303), Anti-Mouse Ly6C PerCP-Cy5.5 (clone AL-21) (BD Biosciences, 560525), Anti-Mouse Siglec-F FITC (clone S17007L) (BioLegend, 155503), Anti-Mouse CD64 FITC (clone X54-5/7.1) (BioLegend, 139316), Anti-Mouse CD40 PE (clone 3/23) (BioLegend, 124609), Anti-Mouse Isotype Ctrl PE (clone RTK2071) (BioLegend, 400408), Anti-Mouse p-IRF3 (S396) Alexa Fluor 488 (clone D601M) (Cell Signaling Technology, 53539S), Anti-Mouse Isotype Ctrl FITC (clone RTK2758) (BioLegend, 400506).

### Identification of lung ILC2 cells.

Lung ILC2 cell identification was performed as described previously (47). Lung tissues were digested in 8 mL RPMI 1640 containing liberase (50 μg/mL) and DNase I (1 μg/mL) for approximately 40 minutes at 37°C. Cell suspensions were filtered through 70 μm cell strainers and washed once with RPMI 1640. For ILC2 cell identification, total lung cell suspensions were blocked with 2.4G2 antibodies and stained with lineage cocktail mAbs: CD3ε (clone 145-2C11) (BioLegend, 100304), CD4 (clone GK1.5) (BioLegend, 100404), CD8α (clone 53-6.7) (Tonbo Biosciences, 30-0081-U500), CD11c (clone N418) (BioLegend, 117304), FceRIα (clone MAR-1) (BioLegend, 134304), NK1.1 (clone PK136) (BioLegend, 108704), CD19 (clone 6D5) (BioLegend, 115504), TER119 (clone TER-119) (BioLegend, 116204), CD5 (clone 53-7.3) (BioLegend, 100604), F4/80 (clone BM8.1) (Tonbo Biosciences, 30-4801-U500), Ly6G (clone RB6-8C5) (Tonbo Biosciences, 30-5931-U500), APC-conjugated streptavidin (BioLegend, 405207), PE-conjugated T1/ST2 (clone DIH9) (BioLegend, 145304), PerCP-Cy5.5-conjugated CD25 (clone PC61) (BioLegend, 102030), V450-conjugated Sca-1 (clone D7) (BD Biosciences, 560653), PE-Cy7-conjugated KLRG1 (clone 2F1/KLRG1) (BioLegend, 138416), APC-Cy7-conjugated CD45, and Fixable Viability Dye eFluor 506.

### Intranuclear and intracellular staining.

Intranuclear staining of Ki-67 and transcription factors was performed with the True-Nuclear Transcription Factor Buffer Set (BioLegend) according to the manufacturer’s instructions. For intracellular cytokine staining, single-cell suspensions from the lungs of mice were prepared with Liberase (50 μg/mL) and DNase I (1 μg/mL). A total of 2 × 10^6^ total live nucleated cells were stimulated in 200 μL RPMI 1640 media containing 10% FBS, Penicillin/Streptomycin (P+S), 2-mercaptoethanol (50 μM), brefeldin A (GolgiPlug, BD Biosciences), and phorbol 12-myristate 13-acetate (30 ng/mL) at 37°C for 3 hours. After surface staining, cells were fixed and permeabilized with BioLegend Cytofix/Perm buffer and further stained intracellularly with anti-mouse IL-5 and IL-13. Dead cells were stained with Fixable Viability Dye eFluor 506 before fixation and permeabilization and excluded during analysis.

### ILC2 cell sorting and culture.

Murine lung ILC2 cells were isolated from Rag1^–/–^ mice treated with IL-33 (0.25 μg/mouse) for 3 consecutive days plus 2 days of resting before processing lung tissues for sorting ILC2 cells with a BD FACSAria cell sorter. The criteria for identifying ILC2 cells are lacking classical lineage markers (CD3ε, CD4, CD8α, CD11c, FceRIα, NK1.1, CD19, TER119, CD5, F4/80, and Gr-1) but expressing markers of CD45 and T1/ST2. The purity of sorted ILC2 cells should be greater than 95%. Sorted ILC2 cells were cultured and expanded in RPMI 1640 media supplemented with 10% FBS, murine IL-2, and IL-7 (all at 10 ng/mL) in 96-well round plates for 6 days before further experiments.

Human ILC2 cells were isolated from peripheral blood of healthy donors or umbilical cord blood samples from healthy full-term births in the Department of Obstetrics and Gynecology of University of Texas Health San Antonio. All human samples were used in compliance with University of Texas Health San Antonio Institutional Review Board. PBMCs or cord blood mononuclear cells were isolated from diluted umbilical cord blood (1:2) by density gradient centrifugation using density gradient medium, Histopaque (MilliporeSigma), and SepMate 50 mL tubes (STEMCELL Technologies, refs. 47, 48). Cells were then washed once with Dulbecco’s PBS-FBS buffer (dPBS, 3% FBS, 1 mM EDTA) and resuspended in dPBS-FBS. Cells were stained with antibodies against CD45, lineage markers (CD3, CD14, CD16, CD19, CD20, and CD56), and ILC2 cell markers CRTH2, CD127, and CD45. Human ILC2 cells were sorted by the BD FACSAria cell sorter as CD45^+^Lin^–^CRTH2^+^CD127^+^ cells. The purity of sorted ILC2 cells was determined to be greater than 95%. Sorted human ILC2 cells were cultured and expanded in RPMI 1640 media supplemented with 10% FBS, rh–IL-2, and rh–IL-7 (all at 50 ng/mL) in 96-well round plates for 6 days before further experiments.

### Culture and treatment of mouse and human ILC2 cells.

Sorted ILC2 cells were cultured with or without 2′3′-cGAMP (5 and 25 μg/mL) in 200 μL complete RPMI 1640 media with or without murine IL-2, IL-7, and IL-33 (all at 10 ng/mL) in 96-well round plates (2000 cells/well) in a 37°C incubator with 5% CO_2_. Three days later, the percentage of IL-5^+^IL-13^+^ cells, Ki-67 expression, and apoptosis of ILC2 cells were analyzed by flow cytometry. Five days later, the number and proliferation of ILC2 cells were analyzed by flow cytometry, and the supernatant was collected for further detecting of IL-5 and IL-13 by ELISA.

Sorted human ILC2 cells were cultured in the complete RPMI 1640 media (200 μL) with or without rh–IL-2, rh–IL-7, and rh–IL-33 (all at 50 ng/mL) in 96-well round plates (2000 cells/well) in a 37°C incubator with 5% CO_2_. The cells were treated with 2′3′-cGAMP, R848, and CpG-A (5 or 25 μg/mL as indicated) for 3 or 5 days. The percentage of IL-5^+^IL-13^+^ cells and the expression of Ki-67 of ILC2 cells were analyzed on day 3 by flow cytometry. On day 5, the number and proliferation of ILC2 cells were analyzed by flow cytometry, and the levels of IL-5 and IL-13 in the supernatants were measured by ELISA.

### Culture and treatment of murine alveolar macrophages.

Murine alveolar macrophages were obtained from BALFs of WT and STING^gt/gt^ mice. Briefly, BALFs were centrifuged at 1500*g* for 7 minutes. Freshly isolated cells were resuspended in complete media (RPMI 1640, 10% FCS, 1% penicillin-streptomycin) with GM-CSF (10 ng/mL, PeproTech) in 96-well round plates (10,000 cells/well) in a 37°C incubator with 5% CO_2_. After 24 hours, the nonadherent cells were discarded; the plates were washed with warm PBS and cultured in fresh complete media with GM-CSF (10 ng/mL). Six days later, these alveolar macrophages from WT and STING^gt/gt^ mice were treated with or without 2′3′-cGAMP (25 μg/mL) in 200 μL complete RPMI 1640 media for another 3 days. Then, the supernatants were collected for further treatment of murine ILC2 cells and detection of mouse IFN-α production by ELISA.

### Depletion of alveolar macrophages using clodronate liposomes.

To deplete lung macrophages, mice were intratracheally administered once with 80 μL clodronate-loaded liposome suspension (Liposoma BV). Control mice were injected with 80 μL PBS-loaded liposomes. To evaluate the efficacy and kinetics of the depletion by clodronate-loaded liposomes, the number of alveolar macrophages in BALF and lung was determined by FACS as shown in Supplemental Figure 9A.

### Lung inflammation and pathology.

Each mouse was intratracheally administered IL-33 or *A*. *flavus* with or without 2′3′-cGAMP. Two days after the last challenge lung tissues were taken and fixed in 4% paraformaldehyde, paraffin embedded, cut into 4 μm sections, and stained with H&E and periodic acid–Schiff (PAS). Complete images of control and treated lungs were obtained digitally using the Aperio Scanscope XT (Aperio). Original magnification ×200 was used for scoring the percentage of PAS^+^ (mucus-producing) bronchial epithelial cells. At least 5 fields were scored to obtain the average for each mouse as previously described (49–51).

### Measurement of pulmonary function.

In the IL-33– and *A*. *flavus*–induced lung inflammation model, changes in mouse pulmonary function after allergen exposure were determined by invasive measurements using the Flexivent system (Scireq). On day 5, the trachea was intubated after anesthetization. The lungs were mechanically ventilated. Indicators of AHR, including airway resistance, elastance, and compliance, were measured after increasing doses (6.25–50 mg/mL) of aerosolized methacholine.

### Statistics.

Statistical analysis was performed using GraphPad Prism 6 software. For comparison of 2 groups, *P* values were determined by unpaired 2-tailed Student’s *t* test, unless otherwise indicated. For comparison of more than 2 groups, 2-way ANOVA was performed. A *P* value of less than 0.05 was considered significant.

### Study approval.

All experiments were performed by following the experimental protocols approved by the IACUC of University of Texas Health Science Center at San Antonio.

## Author contributions

LS, GDB, LY, HHA, and YS performed most experiments. LS, GDB, LY, HHA, EGB, PHD, YS, HZ, DPC, NZ, XZ, YL, and XDL analyzed the data. LS, GDB, LY, HHA, YL, and XDL planned and designed the research. LS, YL, and XDL wrote the manuscript. All authors discussed the results and participated in writing and commenting on the manuscript.

## Supplementary Material

Supplemental data

## Version 1 01/05/2021

In-Press Preview

## Version 2 02/08/2021

Electronic publication
