# Supplementary material for: STING activation in alveolar macrophages and group 2 innate lymphoid cells suppresses IL-33–driven type 2 immunopathology
Source: JCI Insight. 2021 Feb 8;6(3):e143509. doi: 10.1172/jci.insight.143509 (PMC7934858; doi:10.1172/jci.insight.143509)

Figure S1

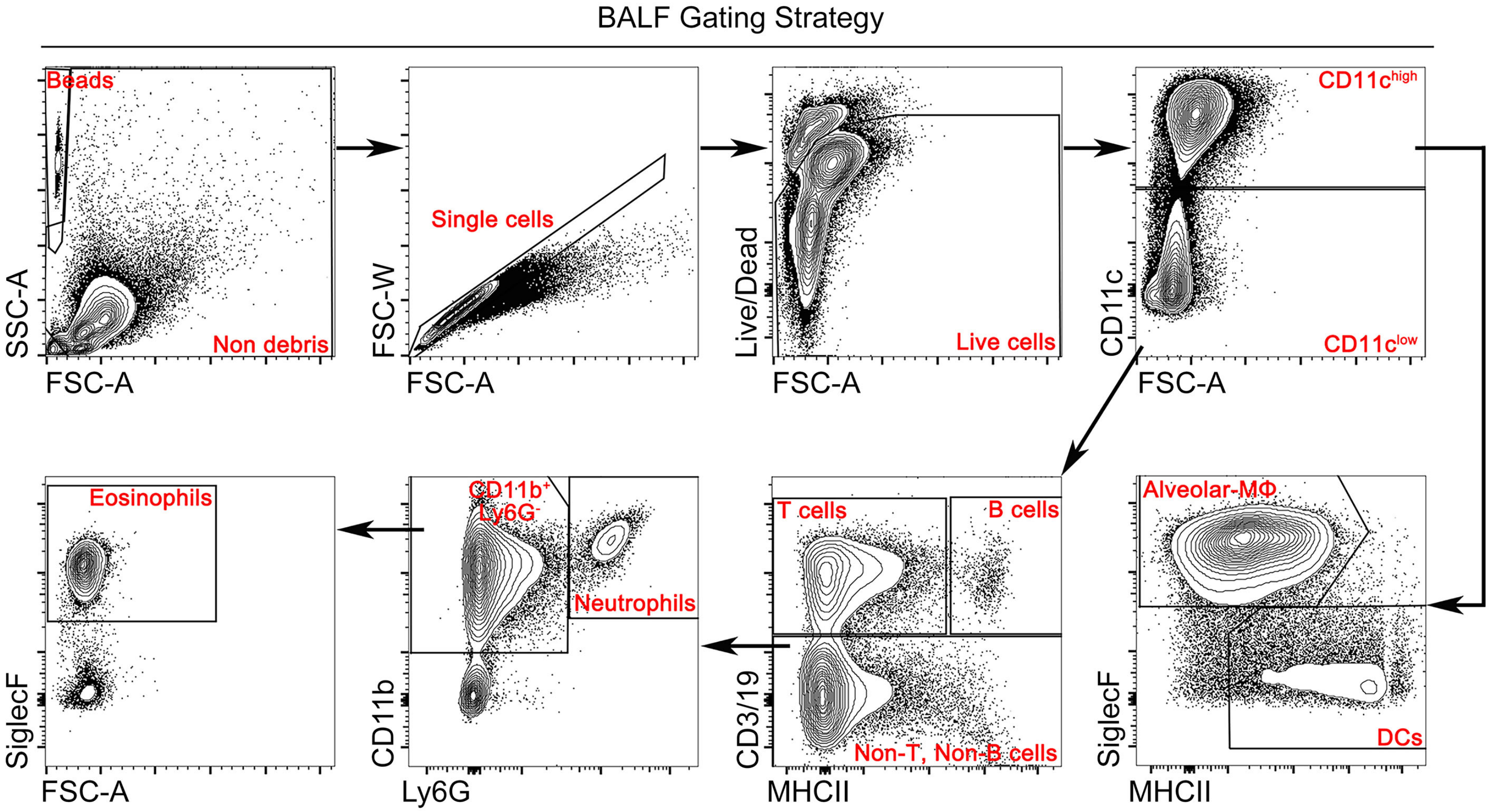

Figure S1. FACS gating strategy for the identification of specific cell types including eosinophils in BALF. Cells recovered from BALF were stained for cell surface markers as indicated. The absolute numbers were calculated based on the reference beads (top panel). Formula (Number of cells per mL): (Total Beads/# events of Beads) x (# event of Sample)/Volume of Tested Sample. (Related to **Figures 1E, 2A, 4B, 5A, 6C, S3A, S5A, S6A, S7B, S8A and S9B**).

Figure S2

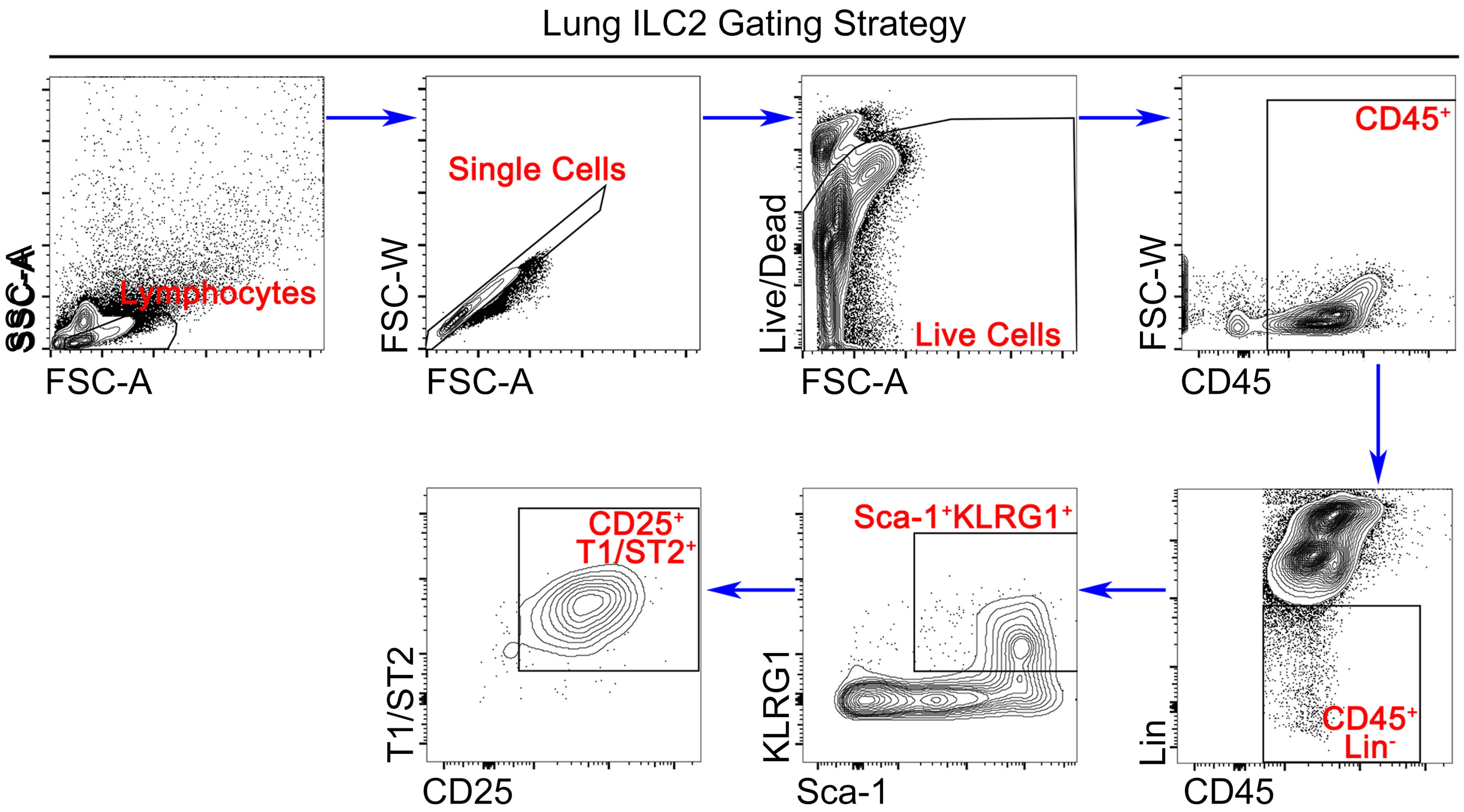

Figure S2. FACS gating strategy for the identification of lung ILC2 cells. Cells recovered from lungs were stained for cell surface markers as indicated. The absolute numbers were calculated based on reference beads (top panel). Formula (Number of cells per mL): (Total Beads/# events of Beads) x (# event of Sample)/Volume of Tested Sample. (Related to **Figures 3, 5C, 6E, S4, S5C, S6C, S7D, S8C and S9D**).

Figure S3 (related to Figure 3B)

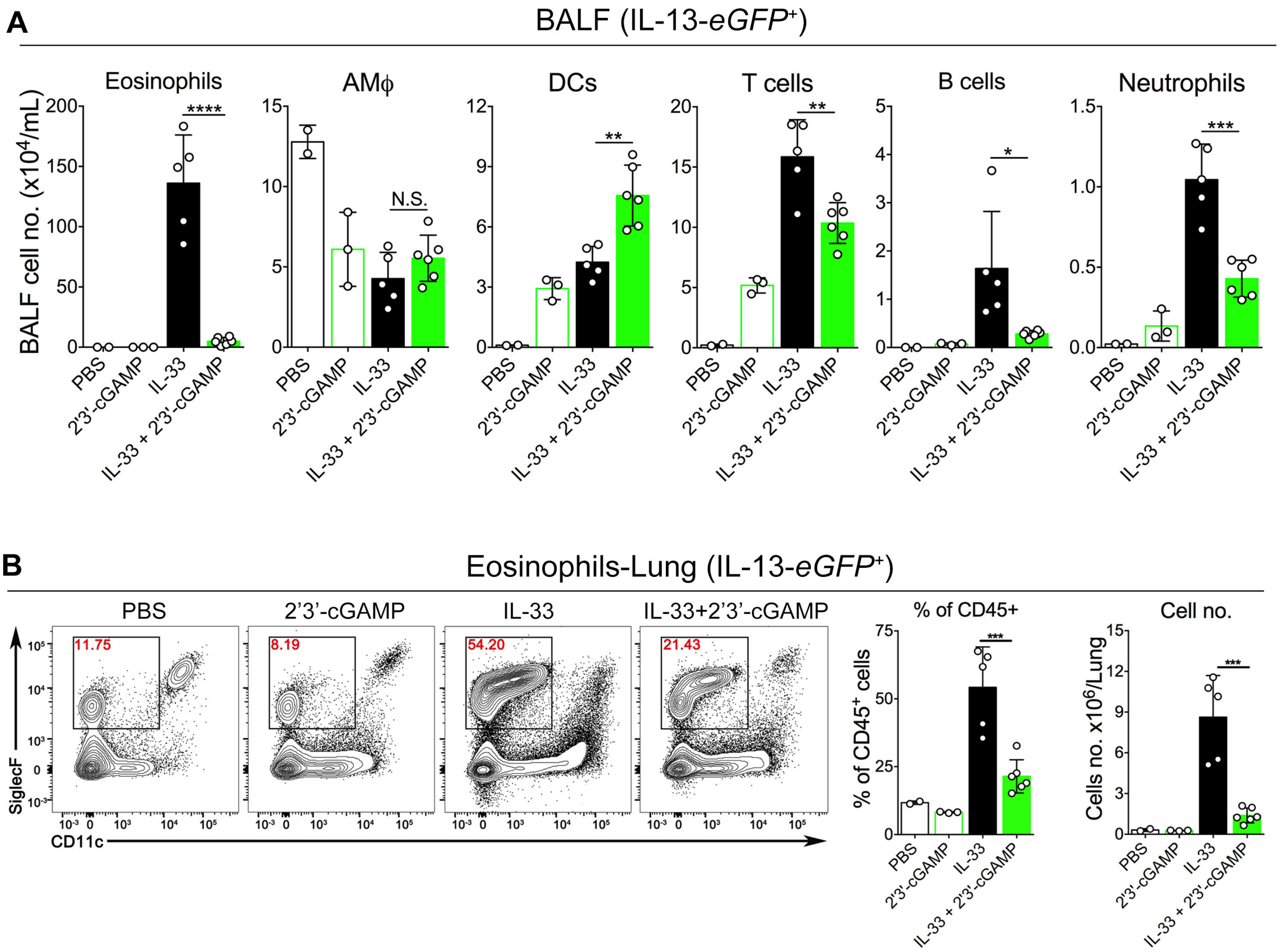

Figure S3. 2'3'-cGAMP inhibits IL-33-induced eosinophilia in IL-13-*eGFP*+ reporter mice. Four groups of mice were treated with PBS, 2'3'-cGAMP, IL-33 or IL-33+2'3'-cGAMP as indicated. (**A**) BALF was collected and analyzed for differential immune cell types. Administration of 2'3'-cGAMP decreased the number of airway eosinophils after exposure to IL-33. (**B**) Administration of 2'3'-cGAMP decreased the percentage and number of lung eosinophils after exposure to IL-33. (n=2-6 per group as indicated with open circles, P value <0.05 was considered statistically significant, unpaired t-test, *** p < 0.001).

Figure S4 (Related to Figure 4)

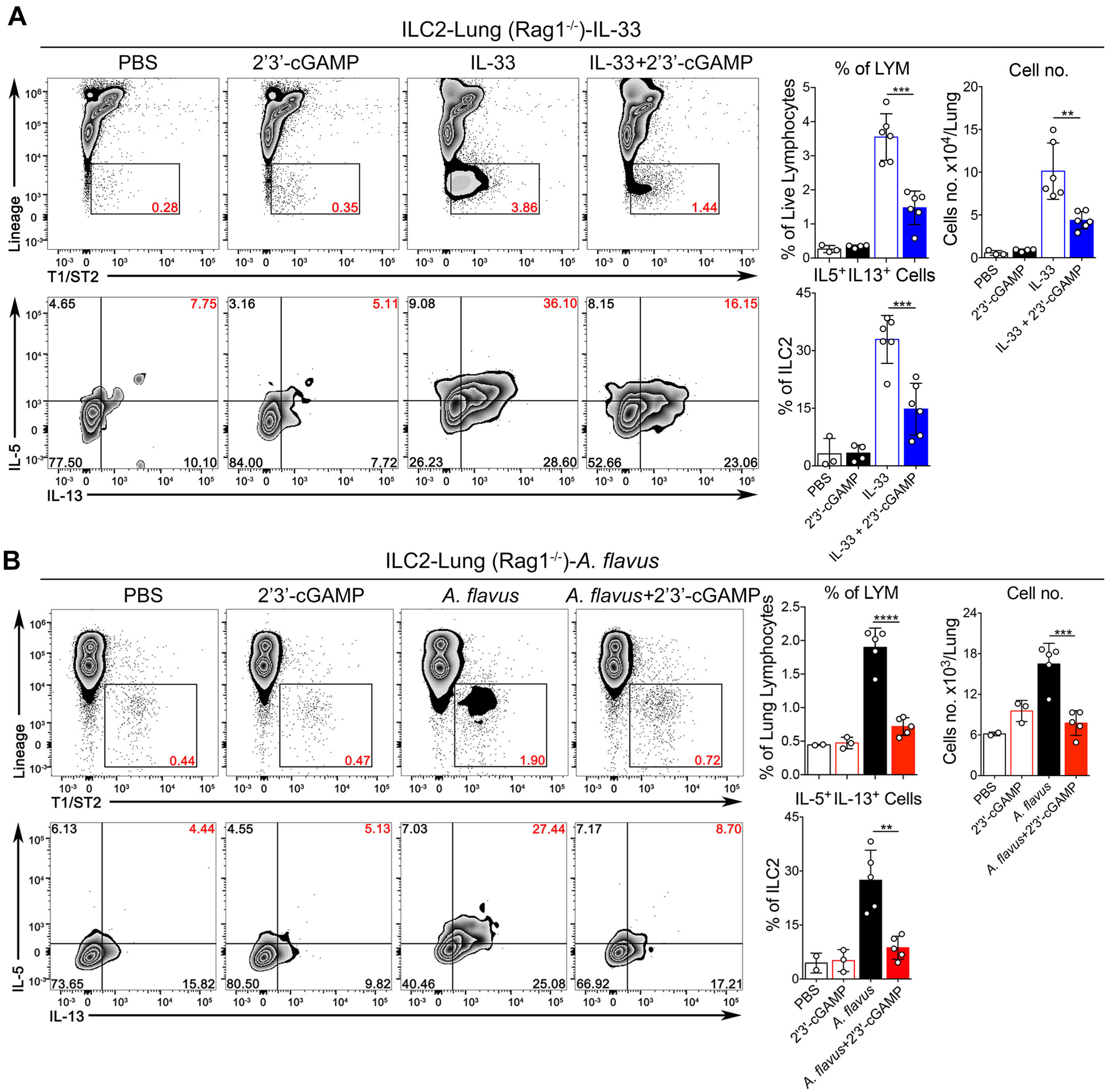

Figure S4. 2'3'-cGAMP inhibits ILC2 activation and proliferation induced by IL-33 or *A. flavus* in Rag1$^{-/-}$ mice. Experimental protocol is same as Figure 4A. Rag1$^{-/-}$ mice were were treated with two kinds of experimental regimens, PBS, 2'3'-cGAMP, IL-33, IL-33+2'3'-cGAMP (**A**) or PBS, 2'3'-cGAMP, *A. flavus*, *A. flavus*+2'3'-cGAMP (**B**). Lung single cell suspensions were prepared and the number of ILC2 cells in lungs were analyzed. In addition, lung cells were stimulated with PMA in cultures as described in the Methods. The percentage of IL5^{+}IL13^{+}-double positive ILC2 cells in lungs were analyzed. (n=2-5 per group as indicated with open circles, P value <0.05 was considered statistically significant, unpaired t-test, ** p < 0.01, *** p < 0.001, **** p < 0.0001).

Figure S5

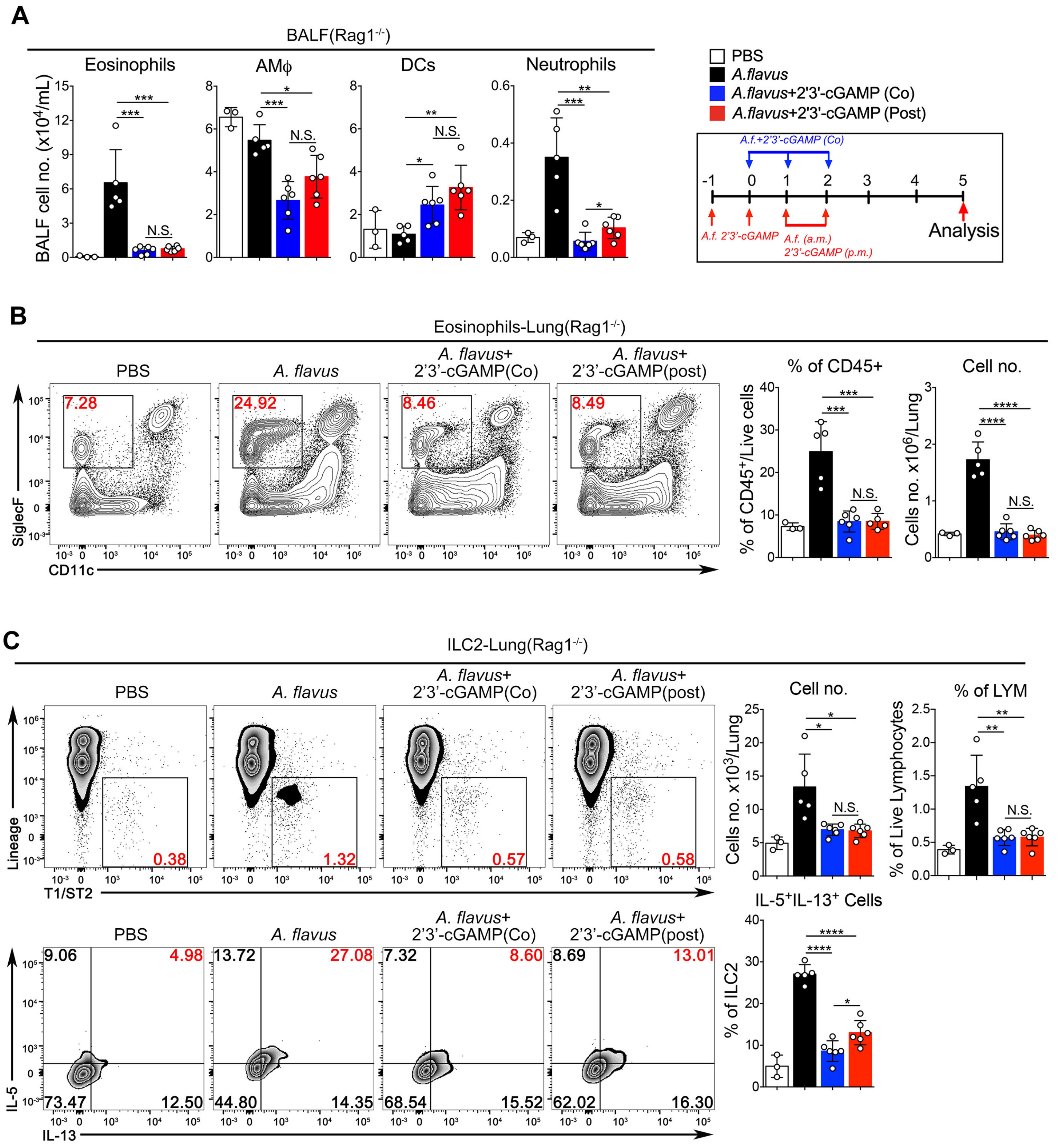
A
BALF(Rag1-/-)
Eosinophils
AMϕ
DCs
Neutrophils
BALF cell no. (x10⁴/mL)
PBS
A.flavus
A.flavus+2'3'-cGAMP (Co)
A.flavus+2'3'-cGAMP (Post)
A.f.+2'3'-cGAMP (Co)
A.f. 2'3'-cGAMP
A.f. (a.m.)
2'3'-cGAMP (p.m.)
Analysis
B
Eosinophils-Lung(Rag1-/-)
PBS
A. flavus
A. flavus+ 2'3'-cGAMP(Co)
A. flavus+ 2'3'-cGAMP(post)
7.28
24.92
8.46
8.49
SiglecF
CD11c
% of CD45+
% of CD45+/Live cells
Cell no.
Cells no. x10⁶/Lung
C
ILC2-Lung(Rag1-/-)
0.38
1.32
0.57
0.58
Lineage
T1/ST2
Cells no. x10³/Lung
% of LYM
% of Live Lymphocytes
IL-5+IL-13+ Cells
% of ILC2
9.06
4.98
73.47
12.50
13.72
27.08
44.80
14.35
7.32
8.60
68.54
15.52
8.69
13.01
62.02
16.30
IL-5
IL-13
N.S.

Figure S5. Co- or post-treatment of 2'3'-cGAMP inhibits *A. flavus*-induced type 2 inflammation in Rag1$^{-/-}$ mice. Four groups of Rag1$^{-/-}$ mice were treated with PBS, *A. flavus*, *A. flavus*+2'3'-cGAMP (Co) and *A. flavus*+2'3'-cGAMP (Post) as indicated**.** BALF were collected and analyzed for differential immune cell types as indicated (**A**). The number and percentage of lung eosinophils (**B**) and ILC2 cells (**C**) were also analyzed. (n=3-6 per group as indicated with open circles, P value <0.05 was considered statistically significant, unpaired t-test, * $p < 0.05$, ** $p < 0.01$, *** $p < 0.001$, **** $p < 0.0001$).

Figure S6

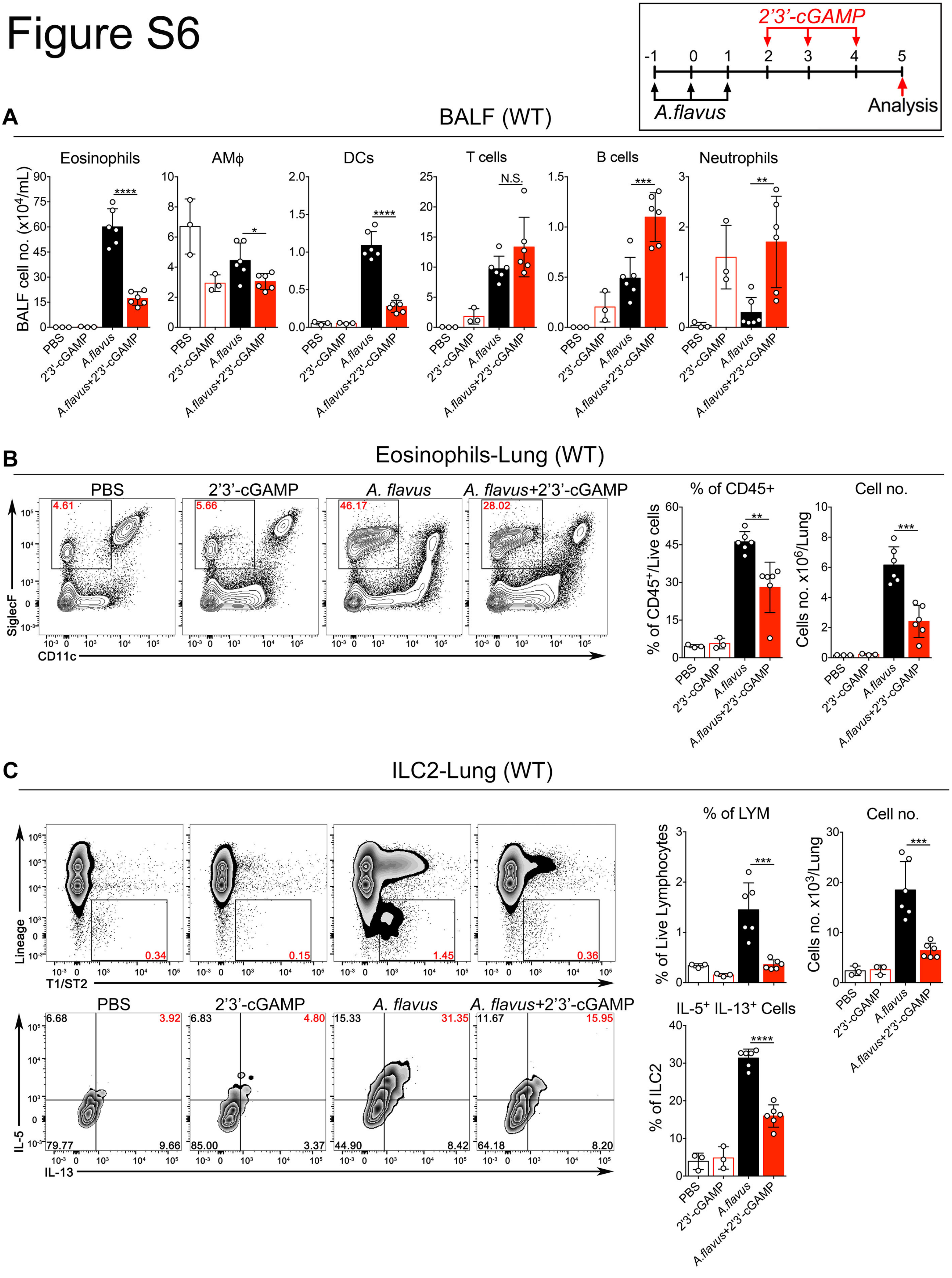

2'3'-cGAMP
-1 0 1 2 3 4 5
A.flavus
Analysis
A
BALF (WT)
Eosinophils
AMϕ
DCs
T cells
B cells
Neutrophils
BALF cell no. (x10^4/mL)
N.S.
PBS
2'3'-cGAMP
A.flavus
A.flavus+2'3'-cGAMP
B
Eosinophils-Lung (WT)
PBS
2'3'-cGAMP
A. flavus
A. flavus+2'3'-cGAMP
4.61
5.66
46.17
28.02
SiglecF
CD11c
% of CD45+
% of CD45+/Live cells
Cell no.
Cells no. x10^6/Lung
C
ILC2-Lung (WT)
Lineage
T1/ST2
0.34
0.15
1.45
0.36
% of LYM
% of Live Lymphocytes
Cell no.
Cells no. x10^3/Lung
IL-5
IL-13
6.68
3.92
79.77
9.66
6.83
4.80
85.00
3.37
15.33
31.35
44.90
8.42
11.67
15.95
64.18
8.20
IL-5+ IL-13+ Cells
% of ILC2

Figure S6. 2'3'-cGAMP can suppress the established type 2 inflammation induced by *A. flavus* in WT mice. Four groups of WT mice were sequentially treated first with PBS or *A. flavus*, then with 2'3'-cGAMP at the indicated time point**.** BALF were collected and analyzed for differential immune cell types as indicated (**A**). The number and percentage of lung eosinophils (**B**) and ILC2 cells (**C**) were also analyzed. (n=3-6 per group as indicated with open circles, P value <0.05 was considered statistically significant, unpaired t-test, * $p < 0.05$, ** $p < 0.01$, **** $p < 0.0001$).

Figure S7

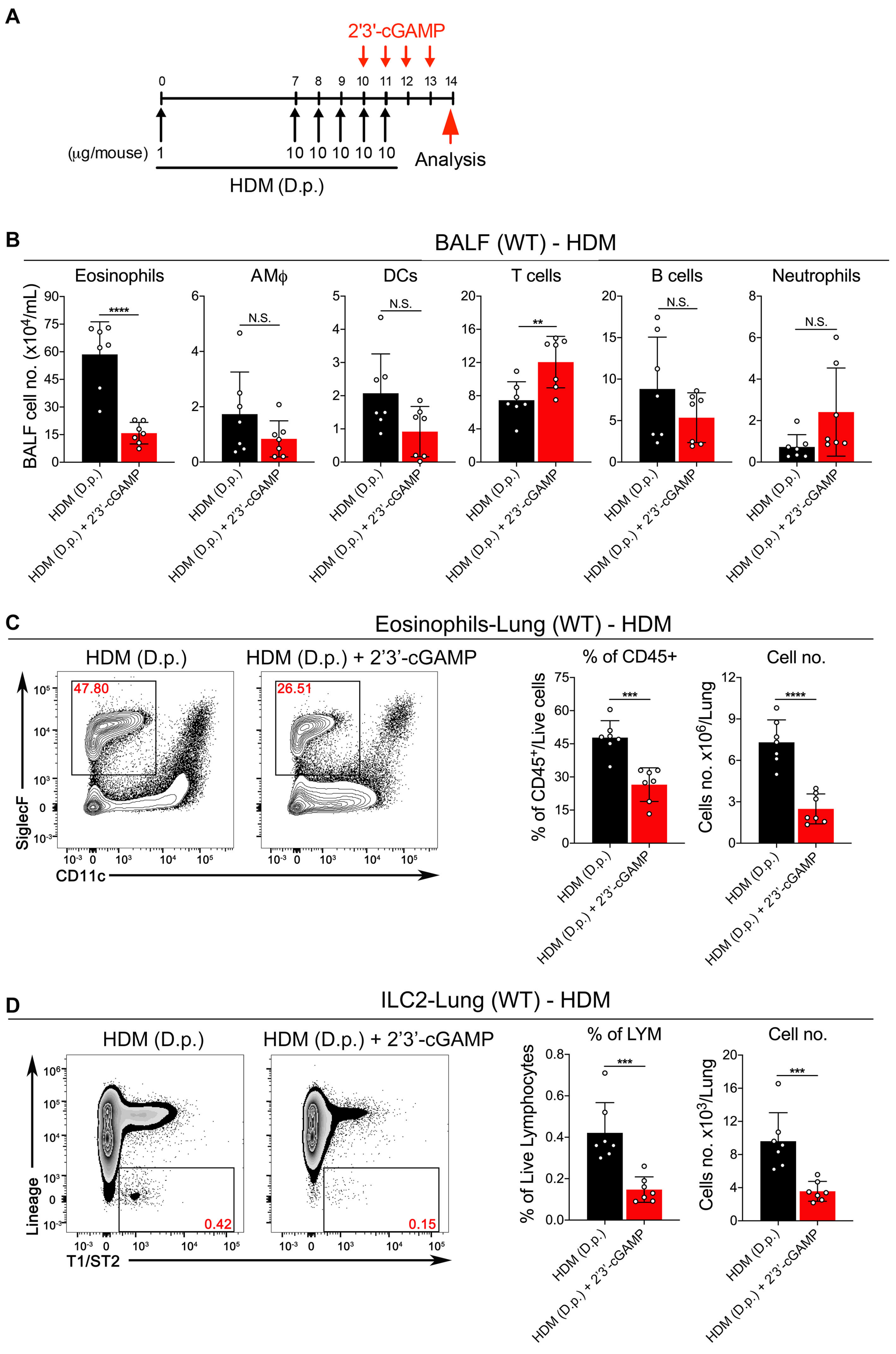
A
2'3'-cGAMP
0 7 8 9 10 11 12 13 14
(µg/mouse) 1 10 10 10 10 10
Analysis
HDM (D.p.)
B
BALF (WT) - HDM
Eosinophils
AMϕ
DCs
T cells
B cells
Neutrophils
BALF cell no. (x10⁴/mL)
****
N.S.
**
HDM (D.p.)
HDM (D.p.) + 2'3'-cGAMP
C
Eosinophils-Lung (WT) - HDM
HDM (D.p.)
HDM (D.p.) + 2'3'-cGAMP
47.80
26.51
SiglecF
CD11c
% of CD45+
Cell no.
% of CD45⁺/Live cells
Cells no. x10⁶/Lung
***
****
D
ILC2-Lung (WT) - HDM
0.42
0.15
Lineage
T1/ST2
% of LYM
% of Live Lymphocytes
Cells no. x10³/Lung

Figure S7. 2'3'-cGAMP can suppress the established type 2 inflammation induced by house dust mites extracts (HDM) in WT mice. (**A**) Experimental protocol showing the animal groups, the corresponding treatment regimen and timeline. Two groups of WT mice were treated with either HDM (D.p.) or HDM (D.p.)+2'3'-cGAMP as indicated. (**B**) BALF were collected and analyzed for differential immune cell types as indicated. The number and percentage of lung eosinophils (**C**) and ILC2 cells (**D**) were also analyzed. (n=7 per group as indicated with open circles, P value <0.05 was considered statistically significant, unpaired t-test, ** $p < 0.01$, *** $p < 0.001$, *** $p < 0.0001$).

Figure S8

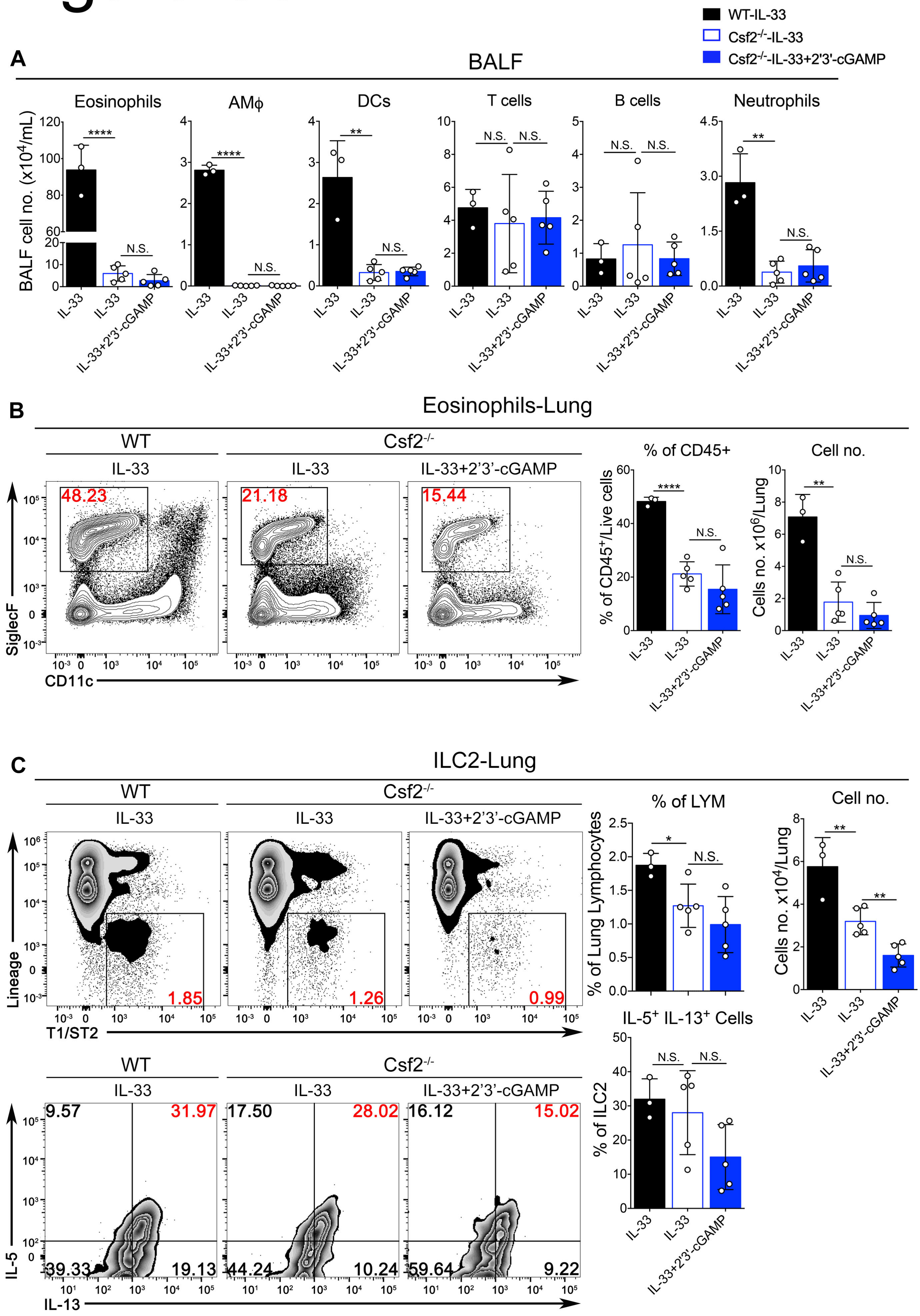

WT-IL-33
Csf2-/--IL-33
Csf2-/--IL-33+2'3'-cGAMP
A
BALF
Eosinophils
AMϕ
DCs
T cells
B cells
Neutrophils
BALF cell no. (x10^4/mL)
N.S.
****
**
IL-33
IL-33+2'3'-cGAMP
B
Eosinophils-Lung
WT
Csf2-/-
IL-33
IL-33+2'3'-cGAMP
48.23
21.18
15.44
SiglecF
CD11c
% of CD45+
% of CD45+/Live cells
Cell no.
Cells no. x10^6/Lung
C
ILC2-Lung
1.85
1.26
0.99
Lineage
T1/ST2
% of LYM
% of Lung Lymphocytes
Cells no. x10^4/Lung
*
IL-5+ IL-13+ Cells
% of ILC2
9.57
31.97
39.33
19.13
17.50
28.02
44.24
10.24
16.12
15.02
59.64
9.22
IL-5
IL-13

Figure S8. The effects of 2'3'-cGAMP on IL-33-induced type 2 inflammation in Csf2$^{-/-}$ mice. (**A**) Groups of WT and Csf2$^{-/-}$ mice as indicated were treated with IL-33 or IL-33+2'3'-cGAMP. BALF was collected and analyzed for differential immune cell types. (**B**) Administration of 2'3'-cGAMP in Csf2$^{-/-}$ mice did not significantly change the percentage and number of eosinophils in lungs after exposure to IL-33. (**C**) The number and percentage of IL5^{+}IL13^{+}-double positive ILC2 cells in lungs of WT and Csf2$^{-/-}$ mice were analyzed. (n=3-5 per group as indicated with open circles, P value <0.05 was considered statistically significant, unpaired t-test, * $p < 0.05$, ** $p < 0.01$, *** $p < 0.001$, **** $p < 0.0001$).

Figure S9

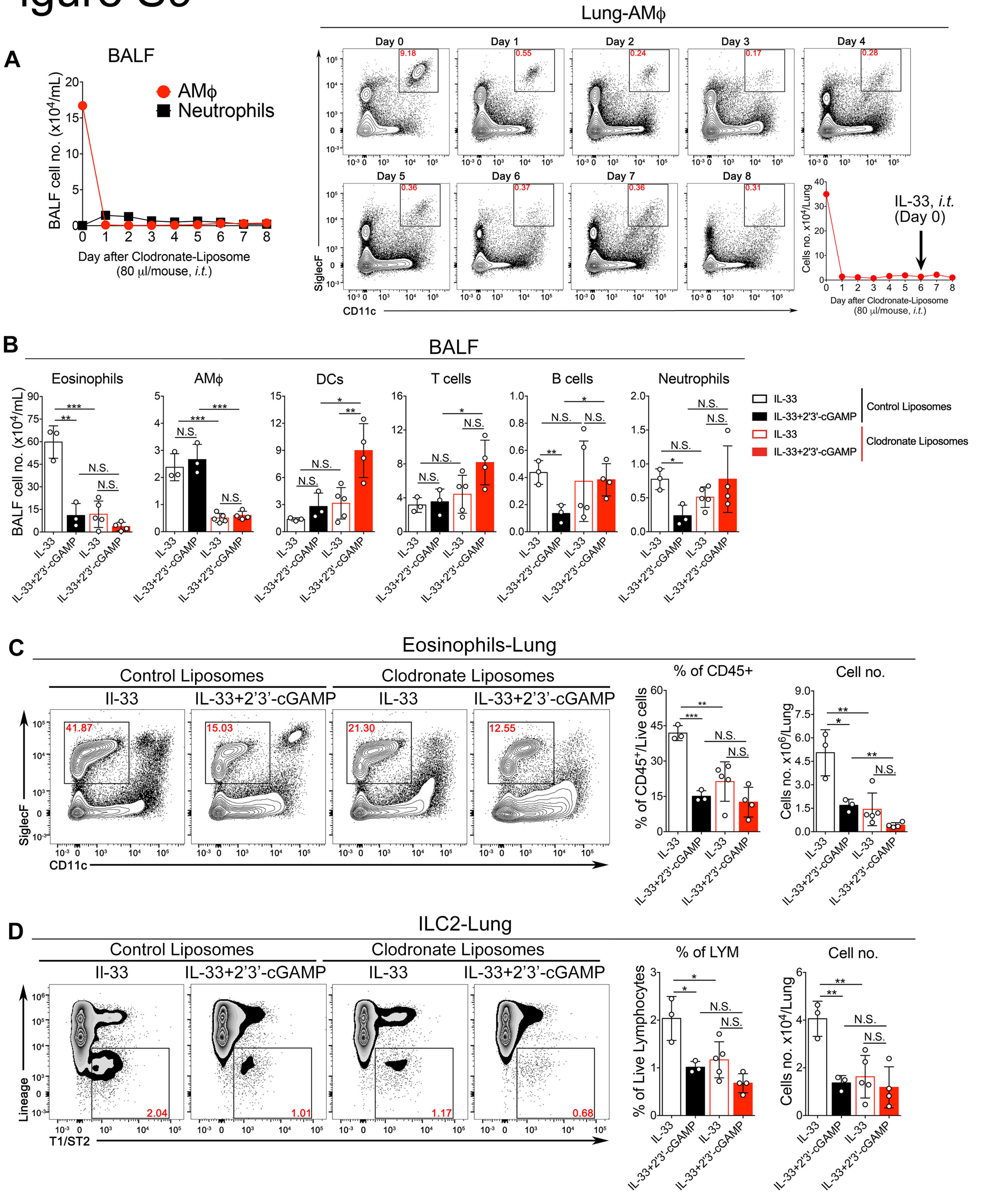
A
BALF
AMϕ
Neutrophils
BALF cell no. (x10^4/mL)
Day after Clodronate-Liposome
(80 µl/mouse, i.t.)
Lung-AMϕ
Day 0
Day 1
Day 2
Day 3
Day 4
Day 5
Day 6
Day 7
Day 8
9.18
0.55
0.24
0.17
0.28
0.36
0.37
0.36
0.31
SiglecF
CD11c
Cells no. x10^4/Lung
IL-33, i.t.
(Day 0)
B
BALF
Eosinophils
AMϕ
DCs
T cells
B cells
Neutrophils
IL-33
IL-33+2'3'-cGAMP
Control Liposomes
Clodronate Liposomes
N.S.
C
Eosinophils-Lung
Control Liposomes
Clodronate Liposomes
Il-33
IL-33+2'3'-cGAMP
IL-33
41.87
15.03
21.30
12.55
% of CD45+
Cell no.
% of CD45+/Live cells
Cells no. x10^6/Lung
D
ILC2-Lung
Lineage
T1/ST2
2.04
1.01
1.17
0.68
% of LYM
% of Live Lymphocytes
Cells no. x10^4/Lung

Figure S9. The effects of 2'3'-cGAMP on IL-33-induced type 2 inflammation in alveolar macrophages-depleted WT mice. (**A**) The kinetics of AMΦ cells in BALF and lung after the treatment with clodronate liposome. (**B**) On day 6 post the liposome treatment, groups of mice as indicated were treated with IL-33 or IL-33+2'3'-cGAMP. BALF was collected and analyzed for differential immune cell types. (**C**) Administration of 2'3'-cGAMP did not significantly change the percentage and number of eosinophils in lungs after exposure to IL-33. (**D**) the number of ILC2 cells in lungs were analyzed. (n=3-5 per group as indicated with open circles, P value <0.05 was considered statistically significant, unpaired t-test, * $p < 0.05$, ** $p < 0.01$, *** $p < 0.001$).

Figure S10

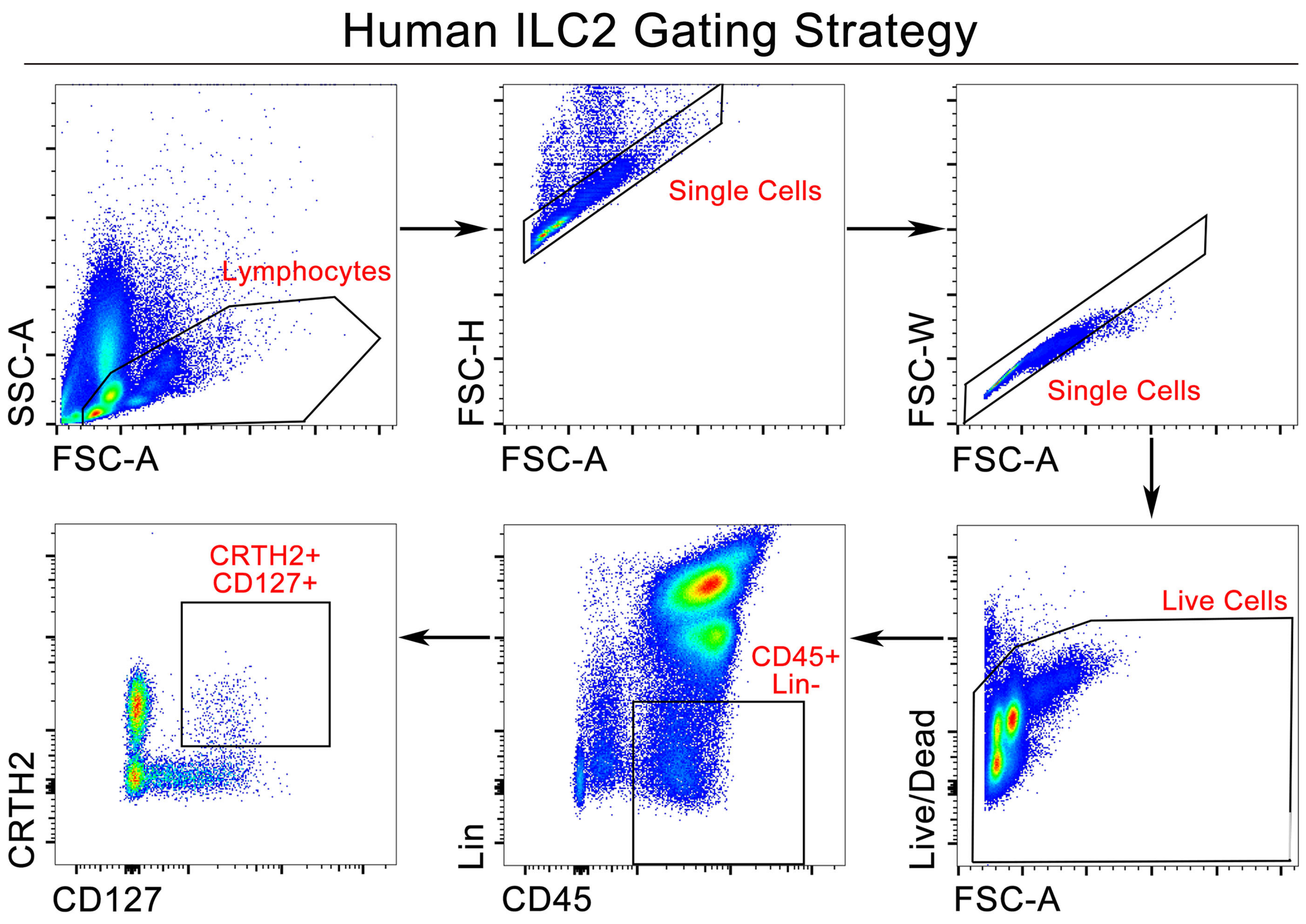

Figure S10. Human ILC2 gating strategy. Human ILC2s were isolated from peripheral blood of healthy donors PBMCs or umbilical cord blood samples CBMCs and stained with antibodies against CD45 and lineage markers as described in the Materials and Methods. Human ILC2s were sorted by the BD FACSAria cell sorter as $CD45^{+}Lin^{-}CRTH2^{+}CD127^{+}$ cells. The purity of sorted ILC2s was determined to be greater than 95%.

Figure S11

A

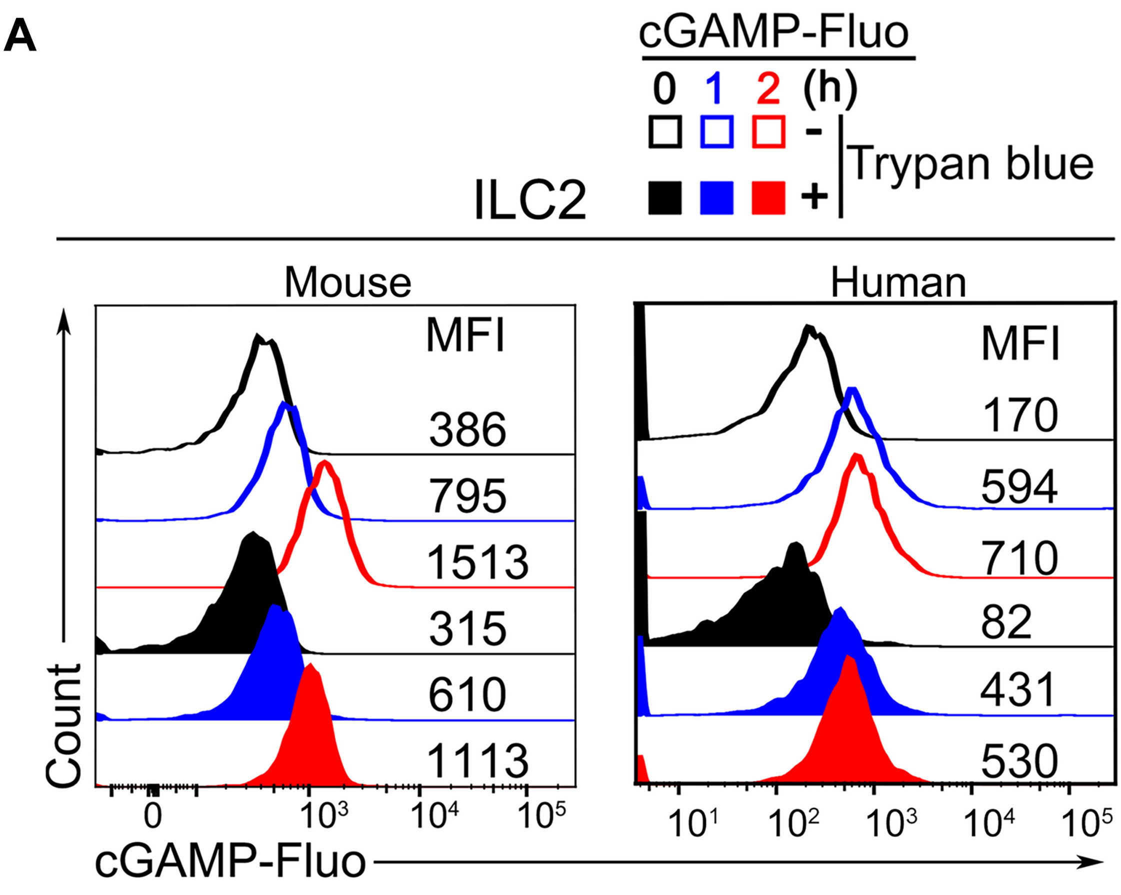

B

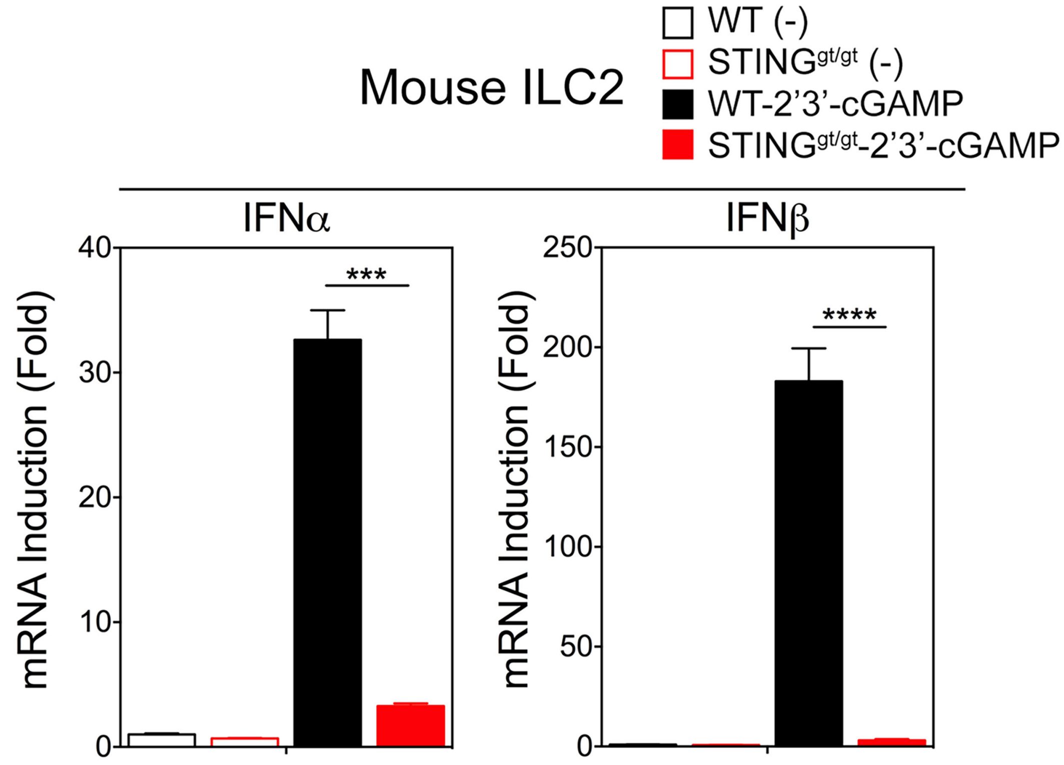

Figure S11. 2'3'-cGAMP can be taken up by IL-33-activated ILC2 cells in vitro. ILC2 cells from human or mice (WT and STING$^{gt/gt}$) were cultured in vitro and treated with fluorescence-labelled or non-labelled 2'3'-cGAMP as indicated. (**A**) The fluorescence-labelled 2'3'-cGAMP (cGAMP-Fluo) can enter both mouse and human ILC2 cells. Trypan blue was used here to quench the surface fluorescence. The result is a representative of three independent experiments. (**B**) RT-qPCR analysis shows that 2'3'-cGAMP can directly stimulate the mRNA expression of the type I interferons in IL-33-activated ILC2 cells in a STING-dependent manner.

03/23/2020

WT / STING$^{gt/gt}$ Mice
/ 2'3'-cGAMP 5ug/mouse

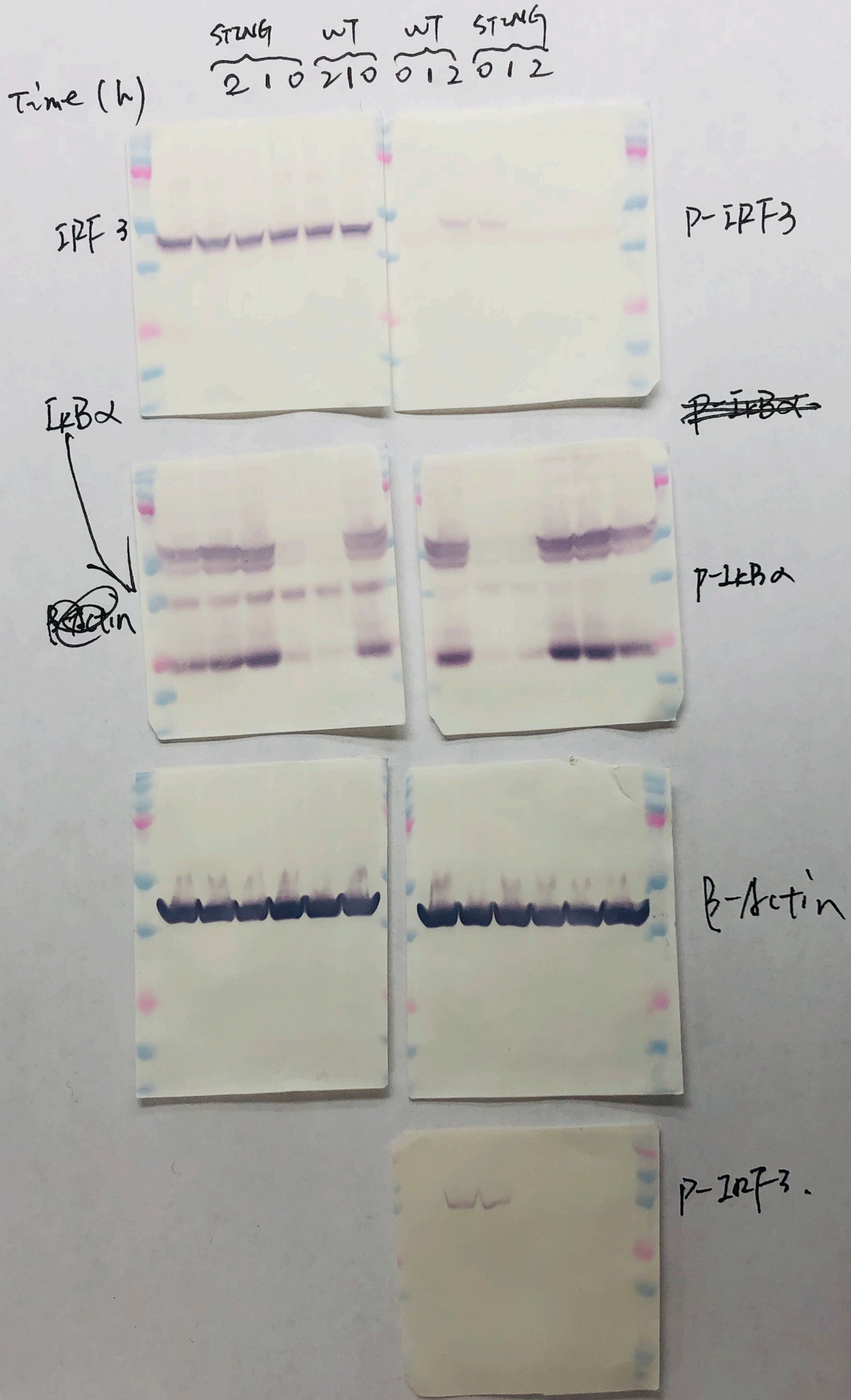

Supplement: Supplemental data [file jciinsight-6-143509-s201.pdf]
